# Spectroscopic Characterization of Thiacarbocyanine Dye Molecules Adsorbed on Hexagonal Boron Nitride: a Time-Resolved Study

**DOI:** 10.1021/acsomega.3c02020

**Published:** 2023-09-20

**Authors:** Anne-Charlotte Nellissen, Eduard Fron, Jonathan B. F. Vandenwijngaerden, Steven De Feyter, Stijn F. L. Mertens, Mark Van der Auweraer

**Affiliations:** † Laboratory for Photochemistry and Spectroscopy, KU Leuven, Chem & Tech, Celestijnenlaan 200F, 3001 Leuven, Belgium; ‡ Department of Chemistry, Energy Lancaster and Materials Science Institute, Lancaster University, Bailrigg, LA1 4YB Lancaster, United Kingdom

## Abstract

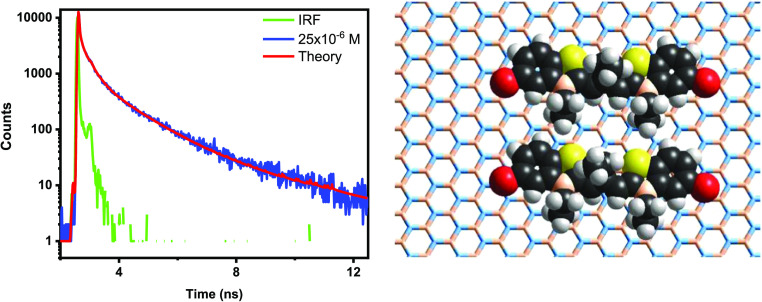

Physisorption on hexagonal boron nitride (hBN) gained interest over the years thanks to its properties (chemically and thermally stable, insulating properties, etc.) and similarities to the well-known graphene. A recent study showed flat-on adsorption of several cationic thiacarbocyanine dyes on hBN with a tendency to form weakly coupled H- or I-type aggregates, while a zwitterionic thiacarbocyanine dye rather led to a tilted adsorption. With this in-depth time-resolved study using the TC-SPC technique, we confirm the results proven by adsorption isotherms, atomic force microscopy, and stationary state spectroscopy combined with molecular mechanics simulations and estimation of the corresponding exciton interaction. The absence of a systematic trend for the dependence of the decay times, normalized amplitudes of the decay components, and contribution of different components to the stationary emission spectra upon the emission wavelength observed for all studied dyes and coverages suggests the occurrence of a single emitting species. At low coverage levels, the non-mono-exponential character of the decays was attributed to adsorption on different sites characterized by different intramolecular rotational freedom or energy transfer to nonfluorescent traps or a combination of both. The difference between the decay rates of the four dyes reflects a different density of the nonfluorescent traps. Although the decay time of the unquenched dyes was in the order of magnitude of that of dye monomers in a rigid environment, it is also compatible with weakly coupled aggregates such as proposed earlier based on the stationary spectra. Hence, the adsorption leads to a rigid environment of the dyes, blocking internal conversion. Increasing the concentration of the dye solution from which the adsorption on hBN occurs increases not only the coverage of the hBN surface but also the extent of energy transfer to nonfluorescent traps. For TDC (5,5-dichloro-3-3′-diethyl-9-ethyl-thiacarbocyanine) and TD2 (3-3′-diethyl-9-ethyl-thiacarbocyanine), besides direct energy transfer to traps, exciton hopping between dye dimers followed by energy transfer to these traps occurs, which resulted in a decreasing decay time of the longest decaying component. For all dyes, it was also possible to analyze the fluorescence decays as a stretched exponential as would be expected for energy transfer to randomly distributed traps in a two-dimensional (2D) geometry. This analysis yielded a fluorescence decay time of the unquenched dyes similar to the longest decay time obtained by analysis of the fluorescence decays as a sum of three of four exponentials.

## Introduction

**Figure 1:**
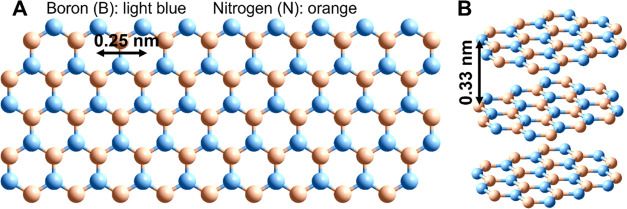
(A) Monolayer hBN and (B) multilayer formation.

Boron nitride nanosheets (two-dimensional (2D) material) are electronic and structural analogs of graphene consisting of alternating sp^2^ hybridized boron and nitrogen atoms linked by strongly dipolar covalent bonds in a hexagonal lattice arrangement.^1−4^ The inter-ring distance between two adjacent borazine units (B_3_N_3_H_6_) is 0.25 nm, while the B–N bond length amounts to 0.15 nm. Hexagonal boron nitride (hBN) is able to form multilayers, where van der Waals interactions keep the layers together at an interlayer distance of 0.33 nm (Figure 1).^1,5^

The surface of boron nitride nanosheets favors physisorption thanks to its chemical structure, allowing noncovalent interactions, such as van der Waals interactions, interactions with π-electrons of the basal plane, and electrostatic interactions with the polarized B–N bonds. The adsorption of various molecules on hBN and on its corresponding nanomesh structure has been studied over the past few years^6−13^ and has also been linked to macroscopic static friction and adhesion.^14^ Using atomic force microscopy (AFM), lamellar structures of hexacontane (C_60_H_122_) molecules on the hBN surface were observed, in agreement with the outcome of molecular mechanics simulations.^10^ Korolkov et al. explored the physisorption of 5,10,15,20-tetrakis(4-carboxylphenyl) porphyrin (TCPP) on hBN.^11^ A square and hexagonal assembly stabilized by in-plane hydrogen bonding between adjacent TCPP molecules and van der Waals interactions between the adsorbed molecules and the hBN substrate could be observed. Bending of the TCPP porphyrin structure occurred due to the aryl side groups, which could not adopt a coplanar structure with the macrocycle, which resulted in a redshift of the fluorescence emission maximum.^11^

**Figure 2:**
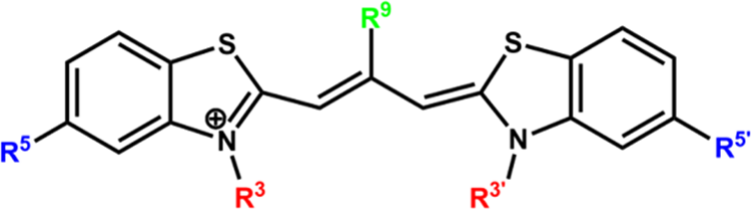
General structure of thiacarbocyanine dyes in their all-*trans* form.

Cyanine dye molecules have also been investigated extensively for their photosensitizing properties^15−19^ and for their potential as fluorescent probes.^20^ Furthermore, depending on the experimental conditions, they can form a large range of well-defined one-dimensional (1D) and 2D aggregates in solution,^21,22^ adsorbed on inorganic surfaces, such as glass,^23^ mica,^24^ silver halides,^17,24^ or TiO_2_,^15,16^^15,16^ incorporated in or adsorbed to Langmuir–Blodgett films^25,26^ or as building blocks for self-assembled polyelectrolyte multilayers where they alternate with layers of a cationic polyelectrolyte such as poly(allylamine) (PAH).^27^ Adsorption of cyanine dyes on substrates, such as mica^24,28,29^ or silver halides,^17,24^ occurs edge-on and leads for meso-substituted carbocyanines generally to the formation of J-aggregates. Also, for adsorption of cationic and zwitterionic carbocyanines to Langmuir films,^25,26^ the obtained excitation and emission spectra show the formation of J-aggregates of closely packed dye molecules, which is only compatible with on-edge adsorption. However, recently, the investigation of the adsorption of several cationic thiacarbocyanine dyes on hBN indicated a flat-on adsorption, leading to the formation of weakly coupled H- or I-aggregates.^12,30^ This conclusion was based on a combination of adsorption isotherms, AFM-determined morphology, excitation and emission spectra with molecular mechanics simulations, and estimation of the corresponding exciton interaction. On the other hand, for a zwitterionic thiacarbocyanine dye, a tilted adsorption on hBN was suggested. To further elucidate the structure and photophysics of the aggregates of the adsorbed dyes on hBN, the fluorescence decays of the same thiacarbocyanine dyes used in the previous stationary study^12^ were obtained at different emission wavelengths and coverages of the hBN substrate. These time-resolved experiments are also expected to give information on the regularity of the adsorbed dye clusters as flaws in the packing geometry of aggregates of thiacarbocyanine dye molecules lead to the formation of nonfluorescent traps, such as observed in self-assembled multilayers of alternating J-aggregates of thiacarbocyanine dyes and the cationic poly(allylamine) (PAH).^27^ Even a small concentration of such traps can quench a large number of surrounding dyes via direct energy transfer or via exciton hopping.^31^ The choice of the dyes (see Figure 2 and Table 1) was based on the presence of extensive knowledge of the photophysics of TDC and THIATS in a large range of different surroundings. The combination of TDC and THIATS allows us to check the effect of the charge: while TDC is a cationic dye, THIATS is a zwitterionic dye with a net negative charge. The difference between TDC and TD2 resides in the chlorine atoms of which the polarizability can enhance van der Waals interactions with the substrate. The difference between TD2 and TD0 is the absence of the 9-ethyl substituent in the latter, which allows for a packing of the adsorbed dyes with interdigitation of the 3- and 3′-substituents and due to less steric crowding leads to a planar structure.^12^

**Table 1:** Abbreviations, Substitution Pattern, Counterions, and Full Name of the Studied Thiacarbocyanine Dyes (Et: ethyl, SulfoPro: sulfopropyl, H: hydrogen, Cl: chlorine, EtSO_4_^–^: tosylate anion, and NH(Et)_3_^+^: triethylammonium ion)

dye	3–3′-R	5–5′-R	9-R	counterion	full name
TDC	Et	Cl	Et	EtSO_4_^–^	5,5′-dichloro-3-3′-diethyl-9-ethyl-thiacarbocyanine
TD2	Et	H	Et	Cl^–^	3-3′-diethyl-9-ethyl-thiacarbocyanine
TD0	Et	H	H	Cl^–^	3-3′-diethyl-thiacarbocyanine
THIATS	SulfoPro	Cl	Et	NH(Et)_3_^+^	5,5′-dichloro-3-3′-disulfopropyl-9-ethyl-thiacarbocyanine

## Materials and Methods

The solvents ethanol (Merck, 99.9%), Milli-Q water (18.2 MΩ cm, total organic carbon <3 ppb), and *n*-heptane (Sigma-Aldrich, 99%) were used without further treatment. The hBN platelets (lateral size <5 μm) used in this study were purchased from Sigma-Aldrich. The thiacarbocyanine dye molecule TDC was a gift from Agfa, while the other thiacarbocyanine dyes TD2, TD0, and THIATS used for this study were synthesized in our research group.^32^

The dye solutions used for adsorption (called “initial dye solutions”) were prepared by diluting a specific volume of a stock solution (ethanol/water (1:1)) of the dye up to 10 mL with EtOH/H_2_O (1:1) in a volumetric flask in order to obtain a solution with the desired concentration. Ten mg of hBN powder was dispersed in 4 mL of the initial dye solution and transferred to conical flacons. Next, the dispersions were centrifuged for 1 h at 4000 rpm. Afterward, the supernatant was separated from the residue and the residue was dispersed with 4 mL of *n*-heptane. These dispersions were spin-coated on a freshly cleaned glass substrate (22 × 22 mm, sonicated for 15 min in Hellmanex solution, Mili-Q water, and isopropanol) for 90 s at 1000 rpm. The samples were sealed with a second glass substrate and then sealed with epoxy glue. Spin-coating of the dye:hBN composites and sealing the samples took place in a glovebox under a nitrogen atmosphere. It was observed that the fluorescence signals from unsealed samples were much weaker, probably due to fluorescence quenching by oxygen or rapid photo-oxidation of the dyes.

In order to quantify the photophysical properties of the adsorbed dyes, we also tried to determine the fluorescence quantum yields using an integrating sphere in a Horiba Jobin Yvon Fluorolog 3 spectrofluorimeter. However, it was not possible to obtain reproducible results due to the low absorbance of the samples.

The fluorescence decays were determined by the time-correlated single-photon counting (TC-SPC) technique.^33−36^ The samples were excited by a Ti:sapphire laser (Tsunami mode-locked, model 3950, Spectra-Physics) pumped by a diode-pumped CW laser (Millenia 10W, Spectra-Physics). The Tsunami output (1000 nm, 2 ps, 82 MHz) was sent into a pulse detector (model 3980, Spectra-Physics) to reduce its repetition rate down to 8.2 MHz and into a frequency doubler/tripler (GWU, Spectra-Physics) to obtain 500 nm excitation pulses. The fluorescence, collected at right angle, was spectrally resolved by a monochromator (Sciencetech 9030, slit width 1 nm) and detected by a microchannel plate photomultiplier tube (MCP-PMT, R3809U-51, Hamamatsu). A time-correlated single-photon timing PC module (SPC 630, Becker & Hickl) was used to obtain the fluorescence decay histogram in 4096 channels. The decays were recorded with 10 000 counts in the peak channel and a channel width of 20 ps per channel. In this way, the time window over which the fluorescence decay was monitored amounted to 12 ns. For some decays recorded at the blue edge of the emission spectrum, it was, due to the low intensity, sometimes preferred to stop the data collection at a lower number of counts.

The excitation was set at the shoulder rather than the maximum of the excitation spectrum of the adsorbed dyes^12^ in order to increase the difference in wavelength between the excitation light and the wavelengths where the fluorescence decays were recorded. In this way, the presence of scattered or reflected excitation light in the observed fluorescence decays could be greatly reduced and even eliminated for most of the experiments. The decays were obtained for several detection wavelengths, mainly near and more to the red than the emission maximum of the dyes, also in order to avoid distortion of the decays by scattered/reflected excitation light.

The decays were analyzed with time-resolved fluorescence analysis (TRFA) software, which is based on the iterative reconvolution of a triple or quadruple exponential decay (eq 1) with the instrumental response function (IRF).
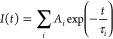
1
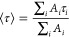
2In eq 1,*A_i_* and τ*_i_* correspond to the amplitude and the decay time of the *i*^th^ component of the decay. The average decay time ⟨τ⟩ is then given by eq 2.^35,37^ The goodness-of-fit was determined by χ^2^ and visual inspection of the residuals and their autocorrelation function. For most samples, χ^2^ was below 1.2. Only for a limited number of samples TD2:hBN 1 × 10^–6^ M (620 nm) and 100 × 10^–6^ M (570 nm), TD0:hBN 1 × 10^–6^ M (580 nm) and 50 × 10^–6^ M (60 nm), and THIATS:hBN 100 × 10^–6^ M (570 nm), it was not possible to obtain values of χ^2^ below 1.2. One should note that the latter decays were mainly recorded at the short wavelength rising edge of the spectra, where the intensity was low, resulting in a poorer signal-to-noise ratio. In order to be able to compare decays of different samples or decays obtained at different emission wavelengths for the same sample, the amplitude of the different components will be expressed as a normalized amplitude α*_i_* (in %) in the tables.
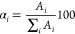
3The contributions in % of each decay component, *p_i_*, to the stationary emission spectrum are given by
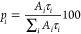
4In order to evaluate the influence of the emission wavelength on the features of the decays, the decays of the same sample obtained at different emission wavelengths were analyzed globally by linking one or more decay times. One should note, however, that there is no physical reason to have three or four components with a different decay time. Actually, nonexponential and stretched exponential decays can also be analyzed as a sum of exponentials.^38^ Hence, one should refrain from associating the different components of the decay with specific excited species or giving physical meaning to the different decay times and the corresponding amplitudes or their contribution to the stationary emission spectrum. For the results shown here, such an interpretation only makes sense for the component with the longest decay time, the component giving the largest contribution to the stationary emission and the average decay time ⟨τ⟩. As an alternative to the analysis as a multiexponential decay, the decays were also fitted to a stretched exponential
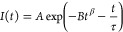
5where β was either kept fixed to 1/3 or allowed to float. In the latter case, the values of β were linked over the different concentrations of the solution from which adsorption occurred.

## Results and Discussion

The earlier determined adsorption isotherms, AFM micrographs, stationary excitation and emission spectra^12^ (see also Figure S1) as well as the fluorescence decays discussed here indicate that while the behavior of TD2 is very similar to that of TDC, TD0 and THIATS show major differences. Therefore, the fluorescence decays of TDC and TD2 are discussed together.

### TDC:hBN and TD2:hBN

**Figure 3:**
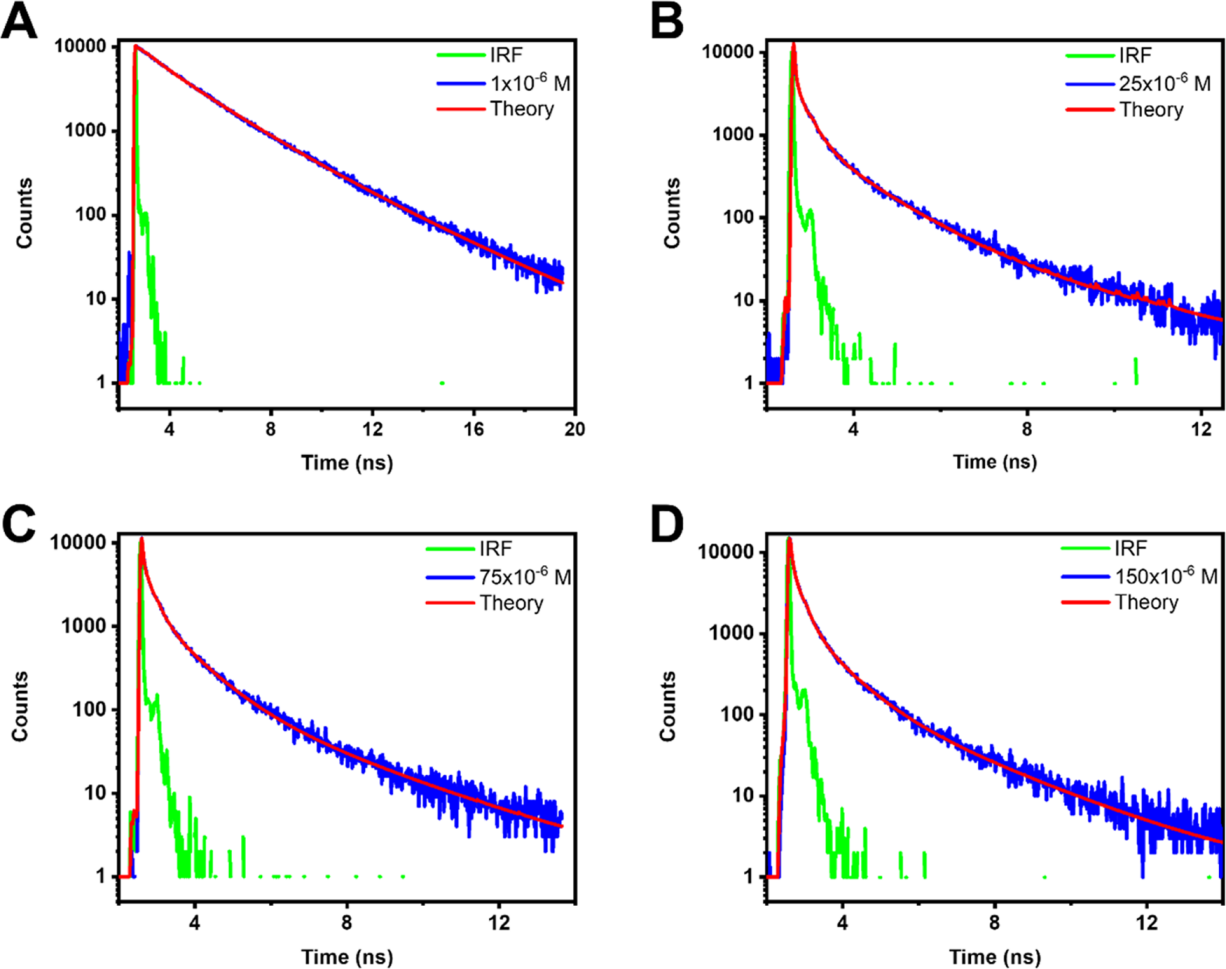
Fluorescence decays of TDC adsorbed on hBN. Excitation occurred at 500 nm, and the decays of the emission were recorded at 580 nm. (A) Adsorption from a 1 × 10^–6^ M solution, (B) adsorption from a 25 × 10^–6^ M solution, (C) adsorption from a 75 × 10^–6^ M solution, and (D) adsorption from a 150 × 10^–6^ M solution.

While for a low coverage, corresponding to an initial concentration of 1 × 10^–6^ M of the TDC solution used for the adsorption, the fluorescence decay is close to single exponential (Figure 3A) with a decay time in the range of 2 to 3 ns, the decay becomes outspoken nonexponential when adsorption occurs from more concentrated solutions leading to a larger coverage (Figure 3B–D). However, between samples made from a dye concentration of 25 × 10^–6^ to 150 × 10^–6^ M, no major changes occur. Visual inspection of the decays shows that under those conditions, the decays also become exponential at long times. While the slope of the tail of the decays does not change much, the amplitude of the slow decaying component is clearly decreased for higher dye concentrations in the solution from which adsorption occurs (Figure 3). Similar results were obtained for TD2 (see Figure S2 in the Supporting Information (SI)). For both TDC and TD2, the decays at 580 nm (close to the maximum of the emission spectrum) (see Figure S1A,B) and at 620 nm (in the red tail of the emission spectrum) had to be analyzed as a sum of four exponentials. Only exceptionally, one of the decay components had no or a negligible amplitude. In this way, it was generally possible to link the decay times recovered at 580 and 620 nm. Although the decays were analyzed as a quadruple exponential decay, one cannot exclude that they represent a stretched exponential decay or a continuous distribution of decay times.^38^

Only for samples prepared for the 10^–6^ M solution of TDC at 580 and 620 nm, an 8 ps component (Table 2) could be recovered. While this component had a large amplitude (α_1_) at 580 nm, its amplitude became very small at 620 nm. Its contribution to the stationary spectrum (*p*_1_) was, however, always smaller than 1%. This component, which was not observed at higher coverages, characterized by a more intense fluorescence, was attributed to scattered/reflected excitation light. Besides this component, also components with decay times of 0.57, 1.87, and 3.29 ns were observed for TDC at the lowest coverage, the latter two contributing together (*p*_3_ + *p*_4_) (Tables 2 and S1) for more than 95% of the stationary emission. For this sample, the ratio α_3_/α_4_ (Table S2) and *p*_4_ are quite similar at 580 and 620 nm. This suggests the presence of only a single emitting species, which is in agreement with the independence of the fluorescence excitation spectra upon the emission wavelength, as reported by us previously.^12^ Except for τ_1_, all decay times are one or several orders of magnitude longer than the decay time of TDC in a nonviscous solution.^32,39^ This is due to the decrease of the mobility of the adsorbed dye molecules prohibiting rotational movements, leading to internal conversion. This agrees with the observation that the fluorescence decay times of cyanine dyes strongly depend upon the viscosity of the environment.^32,39−42^ None of the decay times is however longer than the inverse of the fluorescence rate constant of TDC for which a lower limit of 10^8^ s^–1^ was suggested earlier.^32,42^ The longest decay time (τ_4_) of 3.29 ns is actually close to values observed for the fluorescence decay times of carbocyanines at 77 K or in a viscous environment.^40,41,43^

**Table 2:** Recovered Values of Fluorescence Decay Times (τ_i_) in Nanoseconds and Corresponding Normalized Amplitudes (α_i_) Obtained from the Fluorescence Decays of TDC Adsorbed on hBN from Solutions with Different Initial Concentration (*C*) in mol/L^a^

*C* (×10^–6^ M)	λ_Det_ (nm)	τ_1_ (ns)	α_1_ (%)	τ_2_ (ns)	α_2_ (%)	τ_3_ (ns)	α_3_ (%)	τ_4_ (ns)	α_4_ (%)	*p*_4_ (%)	χ^2^	c (ns)
1	580	0.008	61.6	0.57	5.3	1.87	25.4	3.29	7.7	34	1.00	0.77
	620		0.01		0.0		73.4		26.6	39	1.13	2.25^b^
25	580	0.07	47.1	0.28	40.0	1.14	12.0	3.42	1.0	11	1.10	0.31
	620		55.3		34.4		9.2		1.1	14	1.02	0.27
50	580	0.12	39.1	0.46	39.0	1.22	20.7	3.17	1.2	7	1.08	0.51
	620		32.1		46.8		20.3		0.8	5	1.11	0.52
75	580	0.02	83.1	0.20	12.9	0.84	3.6	2.60	0.4	13	1.07	0.09
	620		46.0		39.1		12.9		2.0	22	1.13	0.25
100	580	0.05	40.8	0.22	39.5	0.74	16.6	1.93	3.1	21	1.12	0.29
	620		33.2		44.3		19.1		3.3	20	1.15	0.32
150	580	0.05	57.6	0.21	34.1	0.78	7.2	2.29	1.1	14	1.04	0.18
	620		43.8		42.6		11.7		1.9	18	1.13	0.24

a *p*_4_ corresponds to the contribution of the component with the longest decay time to the stationary spectrum, while ⟨τ⟩ in ns corresponds to the average decay time. The excitation wavelength was set to 500 nm.

b 1.98 ns when the 0.008 ns component attributed to scattered light is not considered.

In this approach, the decay time of the slowest decaying component (τ_4_) would then correspond to the decay time of unquenched dye molecules (monomers or aggregates), which are rigidified by adsorption on hBN. Although the adsorption isotherm^12^ indicated that for adsorption from a 10^–6^ M solution, only 9% of the surface is covered by adsorbed TDC molecules, the AFM micrographs suggested that under those conditions, the adsorbed dye molecules are already clustering. This is confirmed by the increased Stokes shift compared to that observed for solutions of TDC and TD2, which suggested emission from H-aggregates.^12^ Also Force Field calculations^12^ suggest that clustered adsorbed molecules preferentially form H-type aggregates. Hence, the fluorescence decay time of those aggregates is close to that of cyanine dye monomers in a rigid environment.^40,41,43^ This is quite surprising as it is often observed that the fluorescence of dimers or larger H-aggregates combines a much longer decay time than the monomers in rigid environments^40,41^ with a small fluorescence quantum yield.^30,43−45^ The deviating behavior observed here could be explained by the relatively small exciton coupling suggested by calculations and experiments. Under those conditions, in analogy to what happens in lamellar aggregates of poly(3-hexylthiophene), mixing between different vibronic states of the forbidden and allowed exciton states^30,45−47^ yields an effective fluorescent rate constant, which is not or only slightly depressed by the exciton interaction. The small exciton coupling also leads to a small coherence length of the aggregates. This explains why no significant reduction^21,22,26,27,48,49^ of the FWHM of the emission spectra of the adsorbed aggregated dyes compared to the dyes in solution was observed.^12^

The origin of the 0.57 and 1.87 ns components (τ_2_, τ_3_) can be attributed to either dyes present in an environment with less tight packing, allowing for more rotational mobility, or to quenching of the excited dyes by energy transfer to nonfluorescent traps consisting of aggregated dyes. Fluorescence quenching of similar cyanine dye molecules incorporated in Langmuir films or incorporated in self-assembled films was also attributed to such traps.^25,27,42,48,50−55,56^ Although adsorption from the 10^–6^ M solution leads only to a limited coverage of hBN by the adsorbed TDC, the AFM micrographs indicated under those conditions already clustering of the adsorbed dyes enabling energy transfer to nonfluorescent traps.^12^

At higher coverages, the component with the shortest decay time (τ_1_) becomes the one with the largest amplitude (α_1_), while τ_1_ remains close to the time resolution of the setup. It is unlikely that scattered light plays an important role for these samples as at 580 and 620 nm, similar values for α_1_, the amplitude of this component, are obtained. Although the decay times τ_3_ and τ_4_ decrease systematically upon increasing the dye coverage, the ratio α_3_/α_4_ (Table S2), while two to four times larger than for the sample with the lowest coverage, does not increase further for concentrations of the initial dye solution above 25 × 10^–6^ M. A similar observation can be made for the ratio α_2_/α_3_, which increases by an order of magnitude between 1 × 10^–6^ and 25 × 10^–6^ M, but levels upon further increasing the initial dye concentration. Also, the sum *p*_3_ + *p*_4_, although smaller than for the lowest coverage, does not vary further with increasing coverage and amounts to 55 ± 10%. This means that in analogy to what is observed at the lowest coverage, a large fraction of the stationary emission remains due to the two components with the longest decay times. Also for τ_2_, while about 50% shorter than at the lowest coverage, no systematic change is found upon increasing the concentration of the initial solution beyond 25 × 10^–6^ M. Furthermore, τ_2_ always remains close to the average decay time ⟨τ⟩ with exception of the fluorescence decay at 580 nm of the sample prepared from a 75 × 10^–6^ M solution. The observation that for adsorption from a solution with a dye concentration above 25 × 10^–6^ M no further changes of ⟨τ⟩, τ_2_, α_2_/α_3_, and α_3_/α_4_ are observed agrees with the independence of the maxima of the emission and excitation spectra upon the dye coverage of the hBN substrate when the concentration of the dye solution exceeds 25 × 10^–6^ M.^12^ One should note that at this concentration, already 72% of the hBN surface is covered by the adsorbed dye according to the adsorption isotherm.^12^ In analogy to what was observed at low coverage, also at higher coverage, no systematic dependence of the recovered decay times or amplitudes upon the emission wavelength is observed. This suggests that there is also, at higher coverage, a single emitting species. On the other hand, we observe a redshift of excitation and emission spectra and a 6-fold decrease of the average decay time, mainly attributed the energy transfer to nonfluorescent traps when the initial dye concentration is increased from 1 × 10^–6^ to 25 × 10^–6^ M. This apparently contradicts the AFM data, which suggest that dye clustering and aggregation resulting in exciton interaction already start at initial dye concentration corresponding to a low average coverage.

Possibly at higher initial concentrations of the dye in solution, the nucleation rate of the adsorbed dye clusters is larger, leading to more grain boundaries, diagonal and nondiagonal disorder^49,57^ and defects.^58^ Combined with the possibility of energy hopping in the clusters to the most red-emitting aggregates (exciton diffusion),^57,59−63^ the increased disorder could lead to a small redshift of the emission, while the increased density of defects enhances quenching by energy transfer to nonfluorescent traps. While this hypothesis can explain the redshift of the emission spectra and the faster fluorescence decays deviating more from a monoexponential decay at higher dye coverages, it cannot explain the redshift of the excitation spectra.^12^ The latter suggests that upon increasing the coverage, also a tighter packing of the clustered adsorbed dyes occurs.

If the nonexponential nature of the fluorescence decays is due to energy transfer to randomly distributed, nonfluorescent traps, it should be possible to fit the decay to a stretched exponential (eq 5) with β equal to 1/3 for an adsorbed dye monolayer, which can be considered as a two-dimensional system.^64,65^ In this case, the parameter B in eq 5 corresponds to
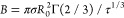
6where σ is the density of traps in nm^–2^, *R*_0_ is the Förster distance for Förster resonance energy transfer (FRET) in nm, and τ is the fluorescence decay time of the unquenched dyes (or dye aggregates) in ns.

When it was attempted to fit the fluorescence decays of TDC adsorbed on hBN at 580 nm for samples prepared with different initial dye concentrations to eq 5 with β fixed to 1/3, acceptable fits were obtained (Figure S3 and Table 3), especially if one considers that compared to a quadruple exponential decay, the space of adjustable parameters has been reduced from 8 to 3.

**Table 3:** Recovered Values of Fluorescence Decay Parameters (B and τ in ns^–1/3^ and ns) and Extracted Values of *σR*_0_^2^ Obtained from the Fluorescence Decays of TDC Adsorbed on hBN from Solutions with Different Initial Concentration (*C*) in mol/L^a^

*C* (×10^–6^ M)	τ (ns)	*B* (ns^–1/3^)	σ*R*_0_^2^	χ^2^
1	2.63	0.38	0.40	1.61
25	32.26	3.90	9.17	1.22
50	2.99	2.82	3.00	1.81
75	20.98	3.77	6.20	1.22
100	6.10	3.77	5.09	1.19
150	11.90	4.40	7.42	1.76

a The excitation wavelength was set to 500 nm and the fluorescence decays were obtained at 580 nm.

While for the lowest dye concentration, the recovered value of τ, the decay time of the unquenched dye, corresponds to what is expected for cyanine dyes in a rigid medium, sometimes much longer and erratically varying values are obtained for the higher dye concentrations. The latter is due to the relatively extensive quenching, leading to a small contribution of unquenched dye molecules at higher concentrations and a correlation between parameters *B* and τ. Although the exact nature of the traps and hence *R*_0_ is not known, one can try to estimate the order of magnitude of σ using a value of 6 nm for *R*_0_. The latter is quite reasonable as for dye molecules values of *R*_0_ lie generally between 4 and 8 nm.^66−68^ This leads to values of σ equal to 1.1 × 10^–2^ nm^–2^ for the lowest dye concentration and values of σ between 8.3 × 10^–2^ and 2.1 × 10^–1^ nm^–2^ for the higher dye concentrations. Although between 1 × 10^–6^ and 25 × 10^–6^ M, σ increases by about an order of magnitude; for dye concentrations above 25 × 10^–6^ M, there is no further trend of σ as a function of the dye concentration or the resulting coverage of the hBN surface by the adsorbed dyes. Although due to the absence of concrete knowledge of *R*_0_, σ is only known within a factor of 2, the trend of σ as a function of dye concentration remains valid, as one can expect that similar traps with similar absorption spectrum are formed at different dye concentrations. Due to the structural similarity of the dyes used here, one can expect that for all dyes, *R*_0_ will be similar. Taking into account that a full monolayer corresponds with a density of 0.52 dye molecules/nm^2^, this means that at low coverages, there is about one trap for each 50 dye molecules, while at higher coverages, this goes to about 1 trap for 2 dye molecules. This value is not realistic as it should lead to major changes in absorption or emission spectra. A possible reason for this discrepancy is that actually, *R*_0_ is rather 8 than 6 nm as found by Kemnitz et al. for adsorbed rhodamine dyes.^66^ According to eq 6, this would lead to values of σ, which are 50% smaller. Another possibility is a combination between quenching by energy transfer and a distribution of decay rates, leading to more complex decays. Kemnitz et al. attributed the recovery of unrealistic high trap densities using eq 5 to the presence of two emitting species with a different fluorescence decay time.^66^ Furthermore, it is possible that other more complex expressions taking into account exciton diffusion have to be used to fit the fluorescence decays.^69−71^

It was also possible to analyze the fluorescence decays of TDC adsorbed to hBN using eq 5 while allowing β to float and keeping it linked over decays obtained for different dye coverages (Table S4). In this case, a value of 0.36 was recovered for β, which is within experimental error the same as 0.33 expected for Förster transfer in 2D.^64,65^ However, the values obtained for τ became more erratic and often much longer than what made physical sense.

For TD2, a qualitative inspection of the fluorescence decays (Figure S2) showed a similar dependence upon coverage. While at low coverages, the observed fluorescence is again mainly due to components with the longest decay times of 2.35 and 3.73 ns, and the two components with a shorter decay time become predominant at higher coverages (Tables S1–S3). Analogous to what is observed for TDC, the amplitudes of the different decay components do not show a systematic behavior upon the emission wavelength in the range 570 to 620 nm. The only difference with fluorescence decays of the adsorbed TDC molecules is that systematically, a larger value is recovered for the two longest decay times τ_3_ and τ_4_, which suggest a smaller density of nonfluorescent traps.

In analogy to what was observed for TDC, it was also possible to fit the decays of TD2 to eq 5 either keeping β constant to 1/3 (Figure S4 and Table S5) or allowing it to float and linking it over decays obtained for different initial dye concentrations (Table S6). When β was kept fixed to 1/3, the recovered values of τ were now limited between 2.22 and 3.46 ns and the recovered values of *B* or σ*R*_0_^2^ were, except for the lowest dye concentration in the initial solution, about 50% lower than those found for TDC. When, in analogy to the estimate made for TDC, *R*_0_ was put equal to 6 nm, σ amounts to 2.8 × 10^–2^ nm^–2^ for the lowest dye coverage and fluctuates between 5.5 × 10^–2^ and 9.1 × 10^–2^ nm^–2^ for the higher dye coverages. For concentrations of the initial dye solution exceeding 25 × 10^–6^ M, in analogy to what was observed for TDC, no systematic variation of σ with the dye concentration is observed. This approach corresponds with the results obtained from the analysis of the fluorescence decays as a sum of exponentials, which also suggested less quenching by the nonfluorescent traps compared to TDC. The less extensive quenching also means that a larger fraction of the molecules decays with a decay time τ, which explains why for TD2 τ can be recovered more accurately than for TDC. When β was allowed to float but kept linked over the different samples corresponding to different coverages, a value 0.53 was obtained for β, which is 60% larger than what is expected for quenching by Förster type energy transfer in a random 2D system. However, in this case, Table S6 shows that the values of τ vary in an erratic way with the dye coverage.

### TD0:hBN

**Figure 4:**
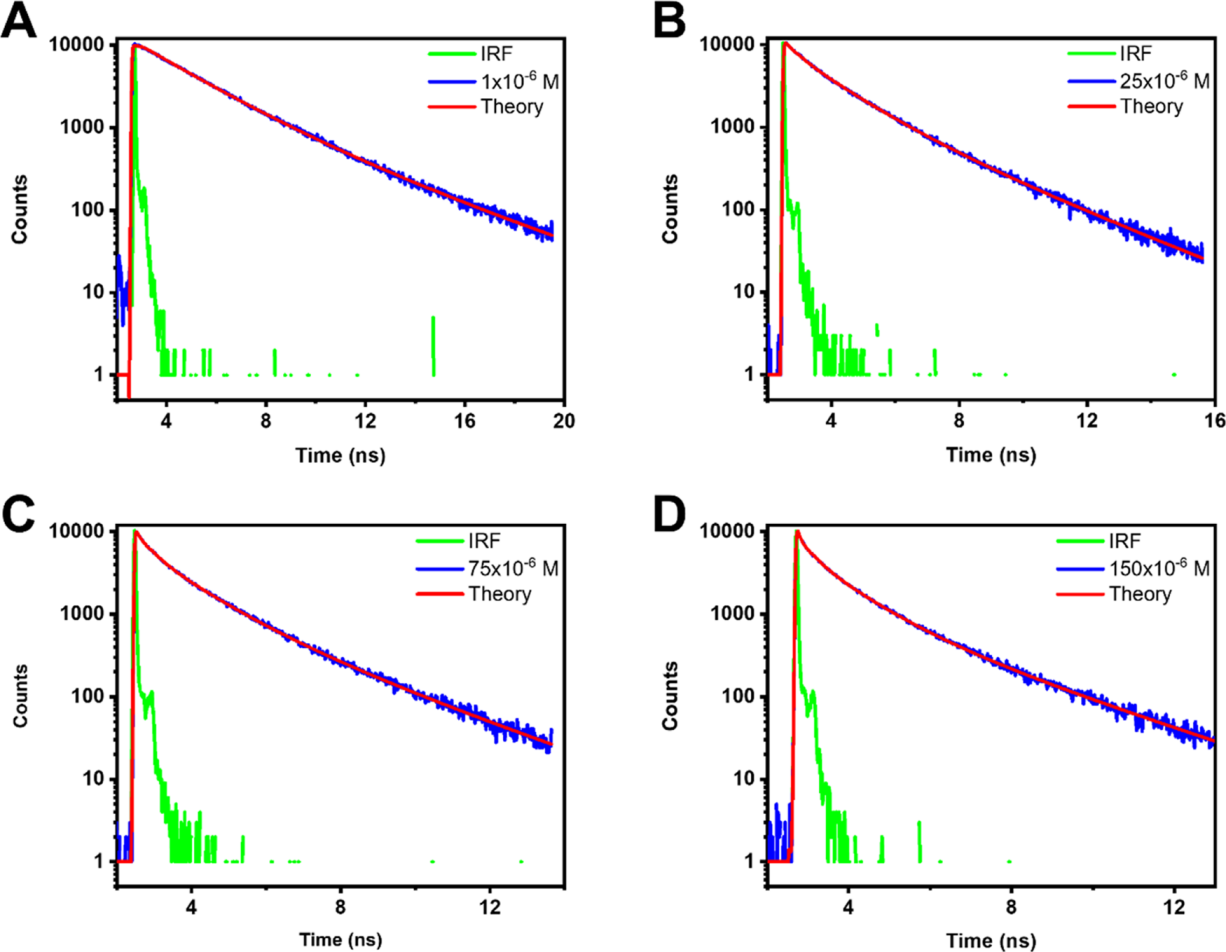
Fluorescence decays of TD0 adsorbed on hBN. Excitation occurred at 500 nm, and the decays of the emission were recorded at 600 nm. (A) Adsorption from a 1 × 10^–6^ M solution, (B) adsorption from a 25 × 10^–6^ M solution, (C) adsorption from a 75 × 10^–6^ M solution and (D) adsorption from a 150 × 10^–6^ M solution.

Visual inspection of the decays of TD0 (Figures 4 and S5) shows that all decays at 600 nm are non-mono-exponential and that this nonexponential character increases when the initial dye concentration is increased from 1 × 10^–6^ to 25 × 10^–6^ M. Upon further increasing the initial dye concentration, no major changes in the features of the decays are observed. While this behavior is analogous to the observations made for TDC and TD2, the decays for all concentrations are slower than those observed for TDC and TD2.

Although the decays of the sample with a low coverage (prepared by adsorption from a 10^–6^ M solution, leading to 6% coverage)^12^ cannot be analyzed globally, there is also no clear trend in the recovered decay parameters (Table 4) throughout the spectrum from the blue edge at 570 nm to the red tail at 620 nm. Hence, in analogy to TDC and TD2, there is no indication that more than one emitting species contributes to the stationary emission spectrum. Similar to the discussion on TDC and TD2, the decay is probably nonexponential rather than multiexponential and, hence, no direct meaning can be given to the decay parameters of the individual components. The component(s), which have the major contribution (*p*_2_ or *p*_3_ varying from 64 to 72%) to the stationary emission, have a decay time of 2.20 to 2.90 ns. At 600 and 620 nm, the rest of the stationary emission is due to component 4 with a decay time of, respectively, 4.23 and 4.27 ns, which contributes 34 and 33% to the stationary emission spectrum. Hence, at 600 and 620 nm, the two slowest decaying components contribute more than 99% of the stationary emission for this sample of TD0. Only at 570 and 600 nm is an extremely fast decaying component (τ_1_) with a decay time of 6 and 0.7 ps, which is much below the time resolution of single-photon timing, is recovered. While this component has a large amplitude (α_1_) at 570 nm, its amplitude is extremely small at 600 nm. This component is due to scattered light, which will have the largest contribution at 570 nm at the short wavelength edge of the stationary emission spectrum and closest to the excitation wavelength. At 570 and 580 nm also a very long-living component with decay times of 9.69 and 8.69 ns and a very small amplitude α_4_ of 0.8 and 1.6% is recovered. These decay times are much longer than the decay times of carbocyanines in rigid media or at low temperatures (3 to 4 ns) or the inverse of the fluorescence rate constant of TD0 (3.6 ns).^32,40−43^ It is unlikely that this component is due to dimers or aggregates as it is only observed 20 and 30 nm to the blue of the emission maximum situated close to 600 nm,^12^ while the latter species would be expected to have a red-shifted emission.^43,47,63^ Considering the appreciable background, especially at 570 nm (Figure 4), this component is probably a combination of the background with the 4.23 and 4.27 ns component recovered at 600 and 620 nm. The longest decay times of the component giving a significant contribution to the stationary emission at 600 and 620 nm (4.23 and 4.27 ns) are close to the decay times of carbocyanines in rigid media or at low temperatures (3 to 4 ns) or the inverse of the fluorescence rate constant of TD0 (3.6 ns).^32,40−43^ Therefore, they are in analogy to the TDC and TD2 samples, probably due to unquenched monomers or aggregates of TD0. As the stationary emission and excitation spectra or AFM micrographs suggest that the TD0 molecules are already clustered and/or aggregated at low coverages, the latter hypothesis is the most likely one. Both the stationary spectra and molecular modeling^12^ suggest that the exciton coupling for TD0 adsorbed on hBN is only 80 cm^–1^. As this small exciton coupling allows mixing of several exciton states,^45−47^ no major changes in the radiative decay rate of the exciton will occur compared to dye monomers. This decay time is significantly longer than the longest decay of 3.29 ns recovered for the low coverage sample of TDC. This can be related to the more planar structure of TD0 related to the absence of steric hindrance by the 9-ethyl substituent. The faster decaying components reflect quenching by energy transfer to nonfluorescent traps analogous to what occurs for TDC and TD2. While for TDC and TD2, which are not completely planar, these components could also be attributed to dye molecules in an environment with less tight packing allowing for more rotational mobility, this is less likely for TD0 where in solution the internal conversion is at least an order of magnitude slower.^32^

**Table 4:** Recovered Values of Fluorescence Decay Times (τ_i_) in Nanoseconds and Corresponding Normalized Amplitudes (α_i_) Obtained from the Fluorescence Decays of TD0 Adsorbed on hBN from Solutions with Different Initial Concentration (*C*) in mol/L^a^

*C* (×10^–6^ M)	λ_Det_ (nm)	τ_1_ (ns)	α_1_ (%)	τ_2_ (ns)	α_2_ (%)	τ_3_ (ns)	α_3_ (%)	τ_4_ (ns)	α_4_ (%)	*p*_4_ (%)	χ^2^	⟨τ⟩ (ns)
1	570	0.006	85.1	0.42	3.5	2.25	10.6	9.69	0.8	22	1.16	0.33
	580	0.75	10.3	1.70	36.3	2.90	51.8	8.69	1.6	6	1.53	2.33
	600	0.0007	7.4	2.20	73.3	4.23	19.3	/	/	34^b^	1.17	2.43
	620	0.20	4.9	2.22	75.6	4.27	19.5	/	/	33^b^	1.09	2.52
25	570	0.01	62.5	0.29	14.3	1.33	17.8	2.77	5.4	34	1.02	0.44
	580		42.1		16.8		29.5		11.6	42	0.97	0.77
	600		0.00		27.2		51.1		21.7	44	1.09	1.36
	620		0.20		22.6		51.2		26.0	49	1.08	1.47
50	570	0.001	54.6	0.25	19.2	1.03	19.8	2.47	6.4	39	1.07	0.41
	580		58.6		15.3		19.3		6.9	42	1.06	0.41
	600		31.1		27.8		30.5		10.3	40	1.49	0.64
	620		40.4		24.8		24.9		9.9	43	1.08	0.56
75	570	0.07	22.8	0.31	35.3	1.21	33.1	2.64	8.8	31	1.08	0.76
	580		12.7		30.8		42.0		14.6	39	0.90	1.00
	600		13.5		33.3		39.3		13.8	38	1.07	0.95
	620		12.0		32.8		38.9		16.3	43	1.05	1.01
100	570	0.07	34.4	0.31	34.0	1.06	26.2	2.86	5.4	28	1.08	0.56
	580		33.1		34.0		28.1		4.8	25	1.05	0.57
	600		38.2		31.7		25.6		4.5	24	1.04	0.53
	620		43.0		30.2		22.6		4.2	25	1.02	0.48
150	570	0.06	31.8	0.28	34.0	1.04	27.3	2.61	6.9	31	1.02	0.58
	580		19.1		31.7		37.9		11.3	38	1.04	0.79
	600		27.8		28.1		33.6		10.5	38	1.06	0.72
	620		29.4		29.4		30.9		10.3	39	1.17	0.69

a *p*_4_ corresponds to the contribution of the component with the longest decay time to the stationary spectrum, while ⟨τ⟩ in ns corresponds to the average decay time. The excitation wavelength was set to 500 nm.

b *p*_3_ instead of *p*_4_.

For samples prepared from solutions with a higher concentration, leading to a more important coverage of the hBN substrate, the longest decay times vary between 2.47 and 2.86 ns, while their amplitude and contribution of the corresponding decay components to the stationary emission spectrum vary between 4.2 to 26% and 24 to 49%, respectively. These parameters show no systematic trend as a function of the analysis wavelength or the further increase of the concentration of the dye solution used for the adsorption beyond 25 × 10^–6^ M. While for these samples, the decay time of this component is close to that recovered for TDC samples prepared from more concentrated dye solutions, both the amplitude and contribution to the stationary spectrum are larger than observed for TDC and TD2, where they are in the range of 0.4 to 3.6% and 5 to 21%. Contrary to what was observed for TDC where the longest decay time continues to decrease upon increasing the dye concentration, no significant trend is observed for the value of τ_4_ recovered for TD0. Both observations indicate less extensive quenching by energy transfer or hopping to nonfluorescent traps possibly due to a smaller density of nonfluorescent defects in the layer of adsorbed TD0. The less outspoken disorder in an adsorbed layer of TD0 compared to TDC is also reflected in the earlier observed AFM micrographs at a higher coverage.^12^

Also, for TD0, it was possible (Figure S7, Tables 5, and S7) to fit the fluorescence decays to eq 5 either keeping β constant to 1/3 (Figure S7, Table 5) or allowing β to float while keeping it linked between the fluorescence decays of samples with different dye coverages (Table S7). In the first case, all recovered values of τ are between 2.58 and 3.34 ns and correspond to the fluorescence decay time expected for a cyanine dye at 77 K or in a rigid medium. Using a value of 6 nm for *R*_0_ application of eq 6 gives a value of σ equal to 6.5 × 10^–3^ nm^–2^ for the sample prepared with an initial dye concentration of 1 × 10^–6^ M and 2.8 × 10^–2^ nm^–2^ for the sample prepared with an initial dye concentration of 25 × 10^–6^ M. For samples prepared using more concentrated dye solutions, σ fluctuates between 4.1 × 10^–2^ nm^–2^ and 6.1 × 10^–2^ m^–2^ and shows no systematic trend. This means, assuming *R*_0_ being the same, that the trap concentration is three times lower than found for TDC and about 40% lower than found for TD2. In this respect, the analysis of the fluorescence decays using eq 5 agrees with the conclusions drawn from the analysis as a sum of exponentials. When β is allowed to float and linked over the different samples, a value of 0.61 is obtained for β, which is nearly twice the value expected for Förster type energy transfer in a random 2D system. Analogous to what was observed for TDC and TD2, the recovered values of τ vary in this case erratically with the dye concentration of the initial dye solution, from which the adsorption occurred.

**Table 5:** Recovered Values of Fluorescence Decay Parameters (B and τ in ns^–1/3^ and ns) and Extracted Values of σ*R*_0_^2^ Obtained from the Fluorescence Decays of TD0 Adsorbed on hBN from Solutions with Different Initial Concentration (*C*) in mol/L^a^

*C* (×10^–6^ M)	τ (ns)	*B* (ns^–1/3^)	σ*R*_0_^2^	χ^2^
1	3.05	*.22*	0.24	2.35
25	2.88	*.97*	1.01	1.87
50	3.08	1.57	1.69	1.69
75	3.00	1.38	1.47	1.51
100	3.34	2.00	2.21	1.83
150	3.28	1.65	1.81	1.68

a The excitation wavelength was set to 500 nm. The fluorescence decays were obtained at 600 nm.

### THIATS:hBN

**Figure 5:**
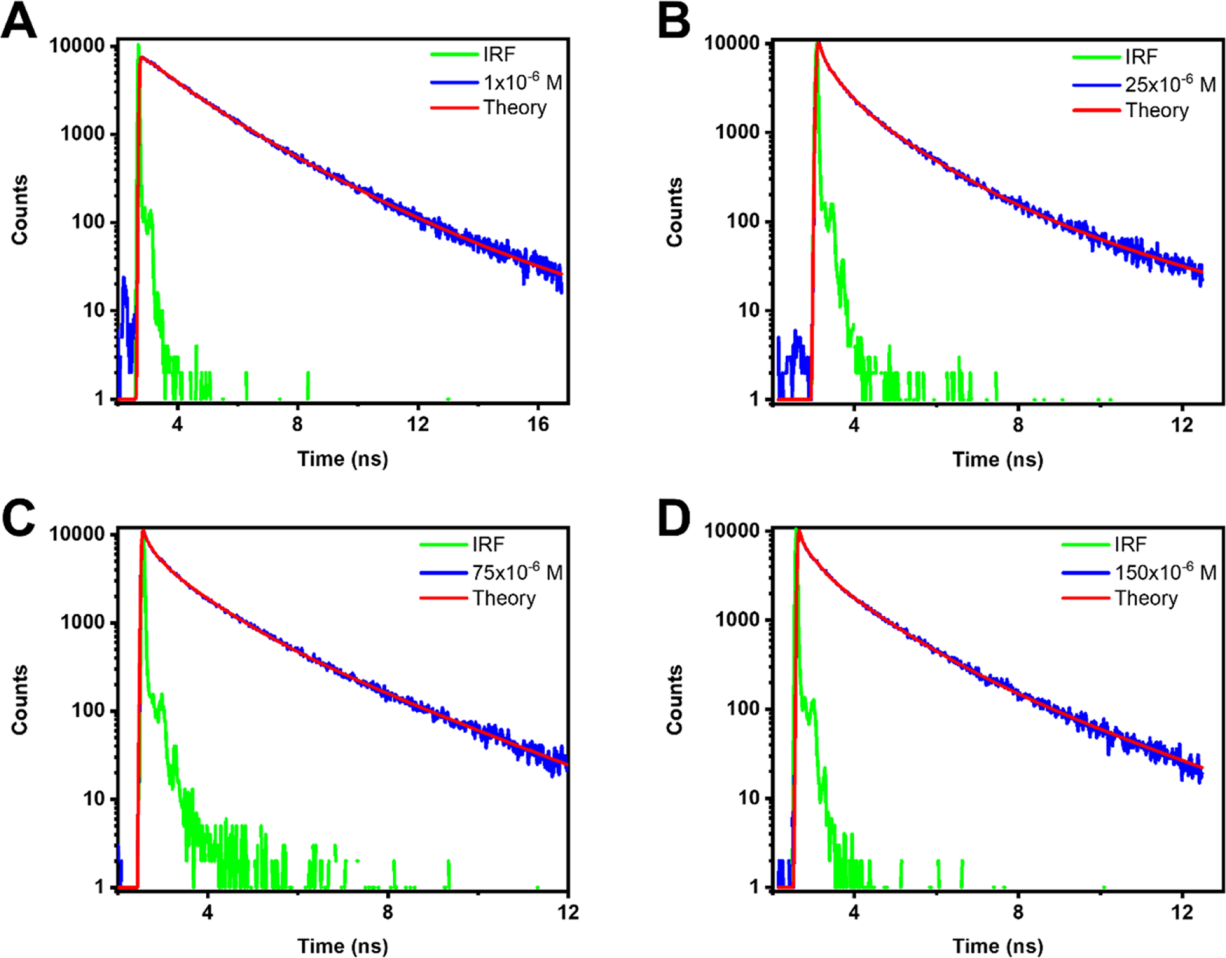
Fluorescence decays of THIATS adsorbed on hBN. Excitation occurred at 500 nm, and the decays of the emission were recorded at 600 nm. (A) Adsorption from a 1 × 10^–6^ M solution, (B) adsorption from a 25 × 10^–6^ M solution, (C) adsorption from a 75 × 10^–6^ M solution, and (D) adsorption from a 150 × 10^–6^ M solution.

Visual inspection of the decays of THIATS (Figure 5) shows that all decays at 600 nm are non-mono-exponential and that this nonexponential character increases when the initial dye concentration is increased from 1 × 10^–6^ to 25 × 10^–6^ M. Upon further increasing the initial dye concentration, no major changes in the features of decays are observed. Similar results were obtained at 580 nm (Figure S8). While this behavior is analogous to the observations made for TDC, TD2, and TD0, the decays are for all concentrations slower than those observed for TDC and TD2 but faster than those observed for TD0.

All decays could be analyzed globally as quadruple exponential decays, while all decay times of the decays obtained at different emission wavelengths were linked (Table 6). Quite surprising, the contribution of the components with the two longest decay times (2.47 and 9.39 ns) decreases for the sample with a low coverage (prepared by adsorption from a 10^–6^ M solution, leading to 3% coverage)^12^ upon increasing the wavelength of the detected emission from 580 to 620 nm. This is also reflected in the average decay time, which decreases from 2.18 to 1.64 ns. Normally, for a condensed packing of chromophores, the wavelength dependence of the fluorescence decays due to spectral diffusion leads to slower decays at longer wavelengths.^57,59−63^ Neither for TDC and TD2 nor for TD0 was such wavelength dependence of the decays observed at a low coverage. This means that for this sample of THIATS molecules adsorbed on hBN, the presence of more than one emitting species cannot be excluded completely. On the other hand, one should note that for the emission at 600 and 620 nm, the maxima of the excitation spectra were nearly identical, suggesting a single emitting species.^12^ The component giving the major contribution to the stationary emission spectrum (contribution *p*_3_ varying from 72% at 580 nm to 55% at 620 nm) has a decay time of 2.49 ns, which, at 580 nm, is close to the average decay time of 2.18 ns. The recovered decay time is of the same order of magnitude as that of the component giving a major contribution to the stationary emission of TD0 adsorbed on hBN and slightly longer than that of the major component of TDC and TD2. However, one should note that for the latter molecules, there is also a component of the decay with decay times of, respectively, 3.29 and 3.73 ns that contributes significantly to the stationary emission. The decay time of the component giving the major contribution to the stationary emission (2.49 ns) is shorter than the decay times of carbocyanines in rigid media or at low temperatures (3 to 4 ns) or the inverse of the fluorescence rate constant of THIATS (upper limit of 10 ns).^32,40−43^ Therefore, this component can in analogy to the TDC, TD2, and TD0 samples be attributed to unquenched monomers or aggregates of THIATS. As the stationary emission and excitation spectra suggest that the THIATS molecules are already aggregated at a low coverage,^12^ the latter hypothesis is the most likely one.

**Table 6:** Recovered Values of Fluorescence Decay Times (τ_i_) in Nanoseconds and Corresponding Normalized Amplitudes (α_i_) Obtained from the Fluorescence Decays of THIATS Adsorbed on hBN from Solutions with Different Initial Concentration (*C*) in mol/L^a^

*C* (×10^–6^ M)	λ_Det_ (nm)	τ_1_ (ns)	α_1_ (%)	τ_2_ (ns)	α_2_ (%)	τ3 (ns)	α_3_ (%)	τ_4_ (ns)	α_4_ (%)	*p*_4_ (%)	χ^2^	⟨τ⟩ (ns)
1	570		not applicable									
	580	0.23	12.5	1.25	20.1	2.47	64.0	9.39	3.4	14.7	1.03	2.18
	600		5.5		50.1		43.9		0.5	3.3	1.00	1.78
	620		11.9		50.4		36.8		0.9	4.5	0.98	1.64
25	570	0.10	42.8	0.39	31.8	1.25	21.4	3.50	4.0	24.5	1.06	0.58
	580		38.3		31.3		27.2		3.2	18.7	0.99	0.62
	600		43.8		31.7		22.1		2.4	15.8	1.08	0.53
	620		51.0		28.5		18.5		2.0	15.3	1.07	0.47
50	570	0.05	38.2	0.24	31.7	0.95	23.7	2.56	6.4	34.1	1.03	0.49
	580		33.5		39.2		23.7		3.6	21.4	1.14	0.43
	600		51.1		33.6		13.8		1.5	14.3	1.21	0.28
	620		45.9		35.5		16.6		2.0	16.2	1.00	0.32
75	570	0.004	79.4	0.19	11.5	0.91	6.6	2.19	2.5	39.1	1.18	0.14
	580		75.6		11.2		9.5		3.7	41.6	1.01	0.19
	600		73.8		12.7		9.9		3.6	40.2	1.15	0.20
	620		76.3		12.0		8.3		3.4	42.0	1.12	0.18
100	570	0.06	53.8	0.24	26.5	0.82	14.8	2.09	4.9	32.2	1.40	0.32
	580		51.4		30.2		14.5		3.9	27.2	0.98	0.31
	600		55.4		30.4		11.4		2.8	22.5	1.04	0.26
	620		65.1		25.7		7.9		1.3	14.33	1.14	0.20
150	570	0.04	66.7	0.27	20.2	1.12	10.2	2.61	2.9	27.3	1.28	0.27
	580		40.7		29.6		23.8		5.9	29.7	0.91	0.52
	600		36.6		31.8		25.8		5.8	27.6	1.08	0.54
	620		53.0		26.1		16.8		4.1	27.0	1.05	0.38

a *p*_4_ corresponds to the contribution of the component with the longest decay time to the stationary spectrum, while ⟨τ⟩ in ns corresponds to the average decay time. The excitation wavelength was set to 500 nm.

The component with the longest decay time (9.39 ns) contributes at 580 nm (at the blue edge of the emission spectrum, see Figure S8) 14.5% to the stationary emission, but its contribution decreases to less than 5% at and beyond the maximum of the emission spectrum (600 and 620 nm). This wavelength dependence is the opposite of what would be expected in the case of exciton diffusion.^57,59−63^ Furthermore, a decay time of 9.39 ns is much longer than the decay times of carbocyanines in rigid media or at low temperatures (3 to 4 ns).^32,40−43^ It is unlikely that this component is due to dimers or aggregates as it is only observed 20 nm to the blue of the emission maximum situated close to 600 nm,^12^ while the latter species would be expected to have a red-shifted emission.^43,47,63^ In analogy to what was discussed for TD0, this component is probably a combination of incompletely corrected dark counts and a component with a decay time of 3 to 4 ns as recovered for TDC, TD2, and TD0. Due to the low counting rate of the sample with the smallest dye coverage and the resulting long data collection time, an appreciable number of dark counts was collected, as can be seen in Figures 5 and S8. The latter effect will be even worse at the blue edge of the spectrum (580 nm), where the intensity is lower than at the maximum, which explains why this apparently long-living component contributes more at 580 nm. In contrast to the other dyes, the sample generated little scattered light, resulting in the absence of a component with a decay time below the time resolution of the setup. The faster decaying components with decay times of 0.23 and 1.49 ns possibly reflect quenching by energy transfer to nonfluorescent traps in analogy to what was concluded for TDC, TD2, and TD0. However, in analogy to TDC and TD2, THIATS is not completely planar,^32^ and these components could also partially be attributed to dye molecules in an environment with less tight packing allowing for more rotational mobility.

Upon increasing the concentration of the dye solution used for adsorption from 1 × 10^–6^ to 50 × 10^–6^ M, the longest decay time decreases to 2.56 ns; however, a further increase of the concentration does not lead to a further decrease of the decay time. In this respect, THIATS resembles TD0 rather than TDC and TD2. A similar observation can be made for the decay time of the component with the largest amplitude (Table 6) or the component giving the largest contribution to the stationary spectrum. This behavior is also reflected in the average fluorescence decay time ⟨τ⟩ (Table 6), which does not decrease further for concentrations of the initial dye solution exceeding 50 × 10^–6^ M. The values of the average decay time in samples prepared from the more concentrated solutions are similar to those found for TDC but shorter than the ones found for TD2 or TD0. Also, for the higher concentrations of the initial dye solution, no systematic dependence of the amplitudes or contributions of the different components to the stationary spectrum on the emission wavelength could be observed. This suggests again that at least for the samples prepared by adsorption of THIATS from a solution with a concentration of 50 × 10^–6^ M or higher, the observed fluorescence is due to the presence of a single emitting species.

In analogy to what was concluded for the three other dyes, the increased decay rate observed for samples prepared from a more concentrated dye solution can be attributed to nonfluorescent defects. In this framework, the defect density is smaller than that in adsorbed layers of TDC but larger than that in adsorbed layers of TD0 or TD2. Furthermore, in contrast to TDC and TD2, where the longest decay continues to decrease upon increasing the initial dye concentration beyond 50 × 10^–6^ M, there is no further increase of the defect density when the concentration of the initial dye solution is increased beyond 50 × 10^–6^ M. The relatively high density of nonfluorescent traps suggested for THIATS on hBN is surprising, as the AFM micrographs obtained earlier^12^ demonstrated a regular morphology without the grainy structures, which were very clear for TDC and less pronounced for TD0.

Also, for THIATS, it was possible (Figure S9, Tables 7 and S8) to fit the fluorescence decays to eq 5 either keeping β constant to 1/3 (Figure S9, Table 7) or allowing β to float while keeping it linked between the fluorescence decays of samples with different dye coverages (Table S7). In the first case, all recovered values of τ are between 2.71 and 4.98 ns and correspond to the fluorescence decay time expected for a cyanine dye at 77 K or in rigid medium. When using a value of 6 nm for *R*_0_, application of eq 6 gives a value of σ equal to 1.9 × 10^–2^ nm^–2^ for the sample prepared with an initial dye concentration of 1 × 10^–6^ M. For samples prepared using more concentrated dye solutions, σ fluctuates between 5.7 × 10^–2^ nm^–2^ and 9.2 × 10^–2^ m^–2^ and shows no systematic trend. This means, assuming *R*_0_ being the same, that the trap concentration is two times lower than found for TDC and about 50% higher than found for TD0 and similar to what is found for TD2. In this respect, the analysis of the fluorescence decays using eq 5 agrees with the conclusions drawn from the analysis as a sum of exponentials. When β is allowed to float and linked over the different samples, a value of 0.48 is obtained for β, which is 60% larger than the value expected for Förster type energy transfer in a random 2D system. Analogous to what was observed for TDC, TD2, and TD0, the recovered values of τ vary in this case erratically with the dye concentration of the initial dye solution from which the adsorption occurred.

**Table 7:** Recovered Values of Fluorescence Decay Parameters (*B* and τ in ns^–1/3^ and ns) and Extracted Values of *σR*_0_^2^ Obtained from the Fluorescence Decays of THIATS Adsorbed on hBN from Solutions with Different Initial Concentration (*C*) in mol/L^a^

*C* (×10^–6^ M)	τ (ns)	*B* (ns^–1/3^)	σ*R*_0_^2^	χ^2^
1	2.71	0.65	0.67	2.33
25	4.11	2.33	2.76	1.55
50	3.80	2.88	3.32	1.31
75	2.72	1.57	1.62	1.39
100	4.98	3.22	4.06	1.14
150	3.18	1.88	2.04	1.21

a The excitation wavelength was set to 500 nm. The fluorescence decays were obtained at 600 nm.

## Conclusions

For all investigated dyes and all coverages (perhaps with the exception of THIATS adsorbed from a 1 × 10^–6^ M solution), no systematic trend for the decay times, the normalized amplitudes, or the contributions of the different components of the decays to the stationary emission spectrum is observed as a function of the emission wavelength. Hence, for all dyes and all coverages, there is most likely a single emitting species. This agrees with the independence of the fluorescence excitation spectra of the adsorbed dyes upon the emission wavelength observed earlier.^12^

Although all decays were analyzed as a triple or quadruple exponential decay, the actual fluorescence decay is probably nonexponential, which is related to energy transfer to nonfluorescent traps.^31^ With the exception of an apparently longer living component with a small contribution mainly at the blue edge of the emission spectra of TD0 and THIATS, the longest decay time observed for samples with a small dye coverage of hBN (<10%), prepared from an initial dye solution with a concentration of 1 × 10^–6^ M, is in a range from 3 to 4.5 ns. Also, analysis as a stretched exponential with β equal to 1/3 yielded decay times of the unquenched dyes between 2.63 and 3.46 ns. This is close to the fluorescence decay time of the dye monomers at low temperatures in a solid or highly viscous environment.^32,40−43^ As this decay time is about 10 times longer than that of TD0 and more than 100 times longer than that of TDC, TD2, or THIATS in a nonviscous solution, this suggests that the emission is due to dye molecules, where internal conversion is blocked by reducing the freedom for rotations around the partially double bonds of the conjugation chain by adsorption to hBN. Taking into account the small exciton interaction extracted from the spectral data^12^ or estimated from the packing of the adsorbed TDC or TD0, suggested by molecular mechanics,^12^ exciton interaction between neighboring adsorbed dye molecules will lead to neither explicit superradiance nor strong depression of the fluorescent rate constant.^59−62^ Therefore, the time-resolved experiments cannot help to discriminate further between H-, J- and I-type aggregates, especially as it was not possible to combine them with reproducible and reliable fluorescence quantum yields.^30^ In this way, the obtained results differ strongly from those obtained for perylenediimides on hBN prepared by physical vapor deposition, where a narrow red-shifted emission band characteristic for J-type aggregates was observed.^13^

However, the similarity of the fluorescence decay time of the rigidized adsorbed dye molecules with that of rigidized dye monomers does not exclude the possibility that aggregation and resulting exciton interaction of the adsorbed dyes occurs at a low dye coverage. This conclusion is compatible with the results of earlier AFM experiments,^12^ suggesting that even upon adsorption from a 1 × 10^–6^ M solution, clustering of the adsorbed dye molecules occurs. Although some exciton interaction between neighboring dyes occurs, this remains too weak to significantly change the excited state decay times or the shape of the emission and excitation spectra as found earlier.^12^

At low coverage, the non-mono-exponential character of the decays can be due to either adsorption of the dye molecule to different sites characterized by a different freedom for intramolecular rotations or energy transfer to nonfluorescent traps as was observed for J-aggregates of TDC and similar dyes adsorbed to Langmuir films or incorporated in build-up polyelectrolyte films or for rhodamine dyes adsorbed to various substrates.^25,39,50,66−69^ The larger values of χ^2^ obtained for the stretched exponential fits for the lowest dye coverage could indicate that besides energy transfer to traps also a distribution of decay rates plays a minor role.^53,72^ At higher coverages, this effect, however, becomes drowned in the quenching by energy transfer to the traps. While it is difficult to prove the relevance of the presence of sites with different rotational freedom, the increase of the decay rate and the more outspoken non-mono-exponential character of the fluorescence decays, when the concentration of the dye solution from which adsorption occurs is increased from 1 × 10^–6^ to 25 × 10^–6^ M and higher, support the hypothesis of fluorescence quenching by energy transfer to nonfluorescent traps. One can expect that higher concentrations of the dye solution lead to higher nucleation rates of the clusters of adsorbed dyes resulting in smaller and less regular clusters with more defects, grain boundaries, and deviations from the ideal packing geometry.^58^ In this case, the differences between the excited state decay rates of the different samples would reflect the density of the fluorescent traps. The slower decays resulting from a smaller density of nonfluorescent energy traps as observed for TD0 compared to THIATS are not reflected in the more homogeneous films of adsorbed THIATS dyes observed by AFM.^12^

Except for a small decrease of the decay time of the component decaying with the longest decay time observed for the analysis of the fluorescence decays of the TDC and TD2 samples as a sum of exponentials, no major changes are observed when the concentration of the dye solution from which adsorption occurs is increased beyond 25 × 10^–6^ M for TDC and TD2 or 50 × 10^–6^ M for TD0 and THIATS, respectively. This makes sense if we look at the adsorption isotherms reported in the literature where we can see that already at an intermediate concentration of 50 × 10^–6^ M for the dye solutions from which the adsorption occurs already, 71 ± 10% of the final coverage is reached for the dyes used.^12^ This also corresponds with the concentration dependence of the emission and excitation spectra, which showed no or little further redshift beyond a concentration of the dye solution of 50 × 10^–6^ M. The decrease of the decay time of the component decaying with the longest decay time at increasing coverage observed for TDC and TD2 suggests that for these dyes, besides direct energy transfer to the traps, exciton hopping between dye dimers followed by energy transfer to the traps also occurs.^69−71^ The occurrence of exciton diffusion could also explain why, especially for TDC, an estimated Förster distance of 6 nm leads to unrealistic high trap densities in the samples with higher dye coverage if only direct energy transfer to the traps is considered.^64,66,69^

## Supporting Information Available

Emission spectra in function of dye concentration, fluorescence decays of TD2 analyzed as a sum of exponentials and as a stretched exponential with β = 1/3, fluorescence decays of TDC, TD0 and THIATS analyzed as a stretched exponential with β = 1/3, fluorescence decays of TD0 and THIATS at shorter emission wavelengths analyzed as a sum of exponentials, parameters of the fluorescence decay of TD2 analyzed as a sum of exponentials and as a stretched exponential with β = 1/3, contributions of the different components of the fluorescence decays of TDC, TD2, TD0 and THIATS analyzed as a sum of exponentials to the stationary emission spectra, ratio of the amplitudes of the different components of the fluorescence decays of TDC, TD2, TD0 and THIATS analyzed as a sum of exponentials, parameters of the fluorescence decays of TDC, TD2, TD0 and THIATS analyzed as a stretched exponential with free β (PDF)
