# Supplementary material for: Spectroscopic Characterization of Thiacarbocyanine Dye Molecules Adsorbed on Hexagonal Boron Nitride: a Time-Resolved Study
Source: ACS Omega. 2023 Sep 20;8(39):35638–52. doi: 10.1021/acsomega.3c02020 (PMC10552479; doi:10.1021/acsomega.3c02020)
Supplement: Supplementary file 1 — ao3c02020_si_001.pdf [file ao3c02020_si_001.pdf]

Spectroscopic Characterization of Thiacarbocyanine Dye Molecules Adsorbed on Hexagonal Boron Nitride: a Time-Resolved Study

AUTHORS

Anne-Charlotte Nellissen[a], Eduard Fron[a], Jonathan B.F. Vandenwijngaerden[a], Steven De Feyter[a], Stijn F.L. Mertens[b,*] & Mark Van der Auweraer[a,*]

AFFILIATIONS

[a]Laboratory for Photochemistry and Spectroscopy, KU Leuven (Chem & Tech, Celestijnenlaan 200F, 3001 Leuven, Belgium)

[b]Department of Chemistry, Energy Lancaster and Materials Science Institute, Lancaster University (Bailrigg, LA1 4YB Lancaster, United Kingdom)

*Corresponding authors: s.mertens@lancaster.ac.uk or stmerten@gmail.com & mark.van-derauweraer@kuleuven.be

SUPPORTING INFORMATION

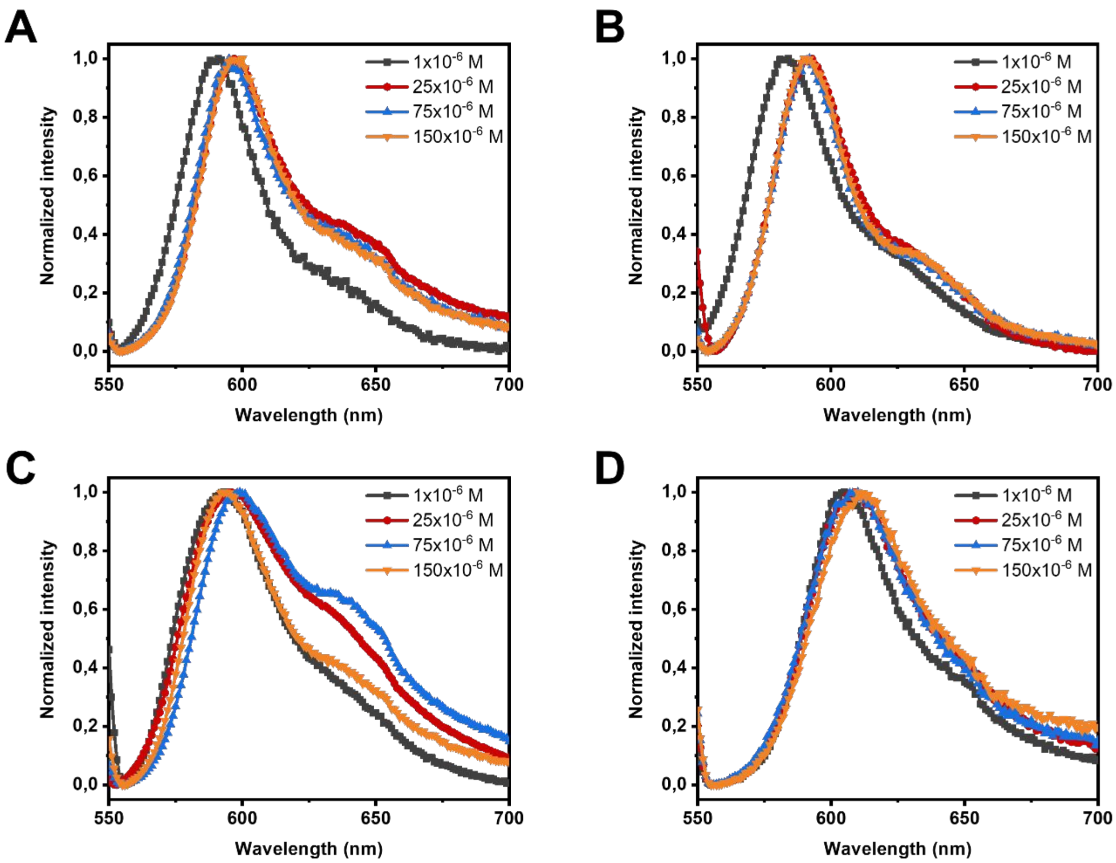

Figure S1 Emission spectra of A) TDC B) TD2 C) TD0 and D) THIATS adsorbed on the surface of hBN with increasing initial dye solution concentration. Excitation wavelength was 525 nm.

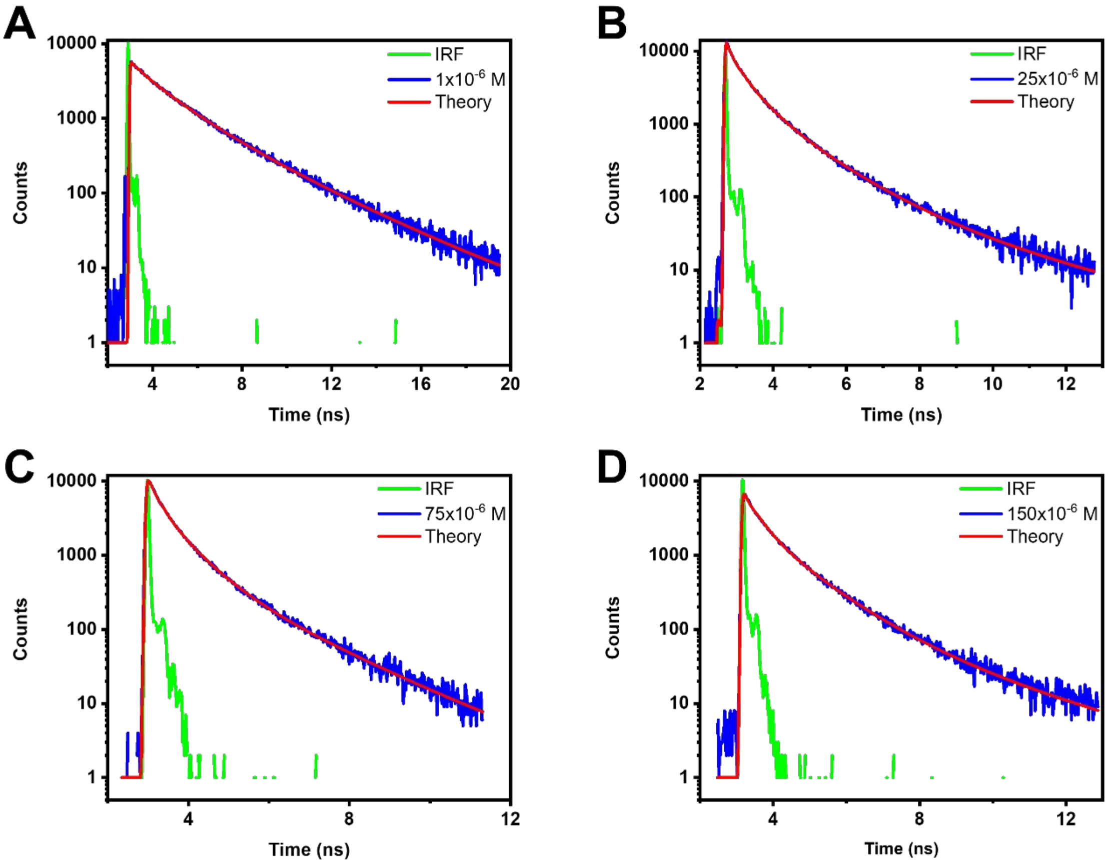

Figure S2 Fluorescence decays of TD2 adsorbed on hBN analyzed as a sum of exponentials. Excitation occurred at 500 nm and the decays of the emission were recorded at 580 nm. A) adsorption from a $1x10^{-6}$ M solution, B) adsorption from a $25x10^{-6}$ M solution, C) adsorption from a $75x10^{-6}$ M solution and D) adsorption from a $150x10^{-6}$ M solution.

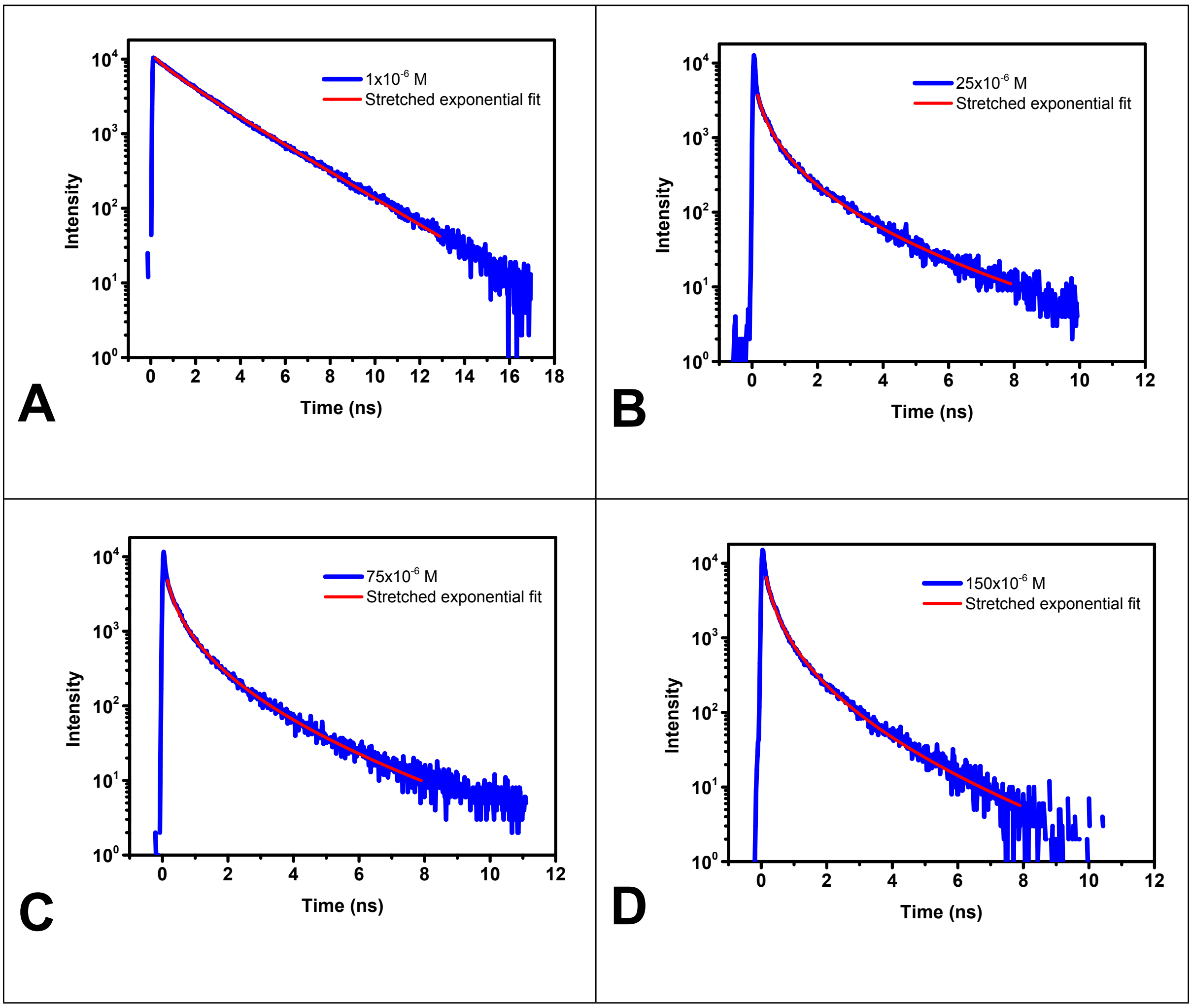

Figure S3 Fluorescence decays of TDC adsorbed on hBN analyzed according to equation 5 with β fixed to 1/3. Excitation occurred at 500 nm and the decays of the emission were recorded at 580 nm. A) adsorption from a $1x10^{-6}$ M solution, b) adsorption from a $25x10^{-6}$ M solution, c) adsorption from a $75x10^{-6}$ M solution and d) adsorption from a $150x10^{-6}$ M solution.

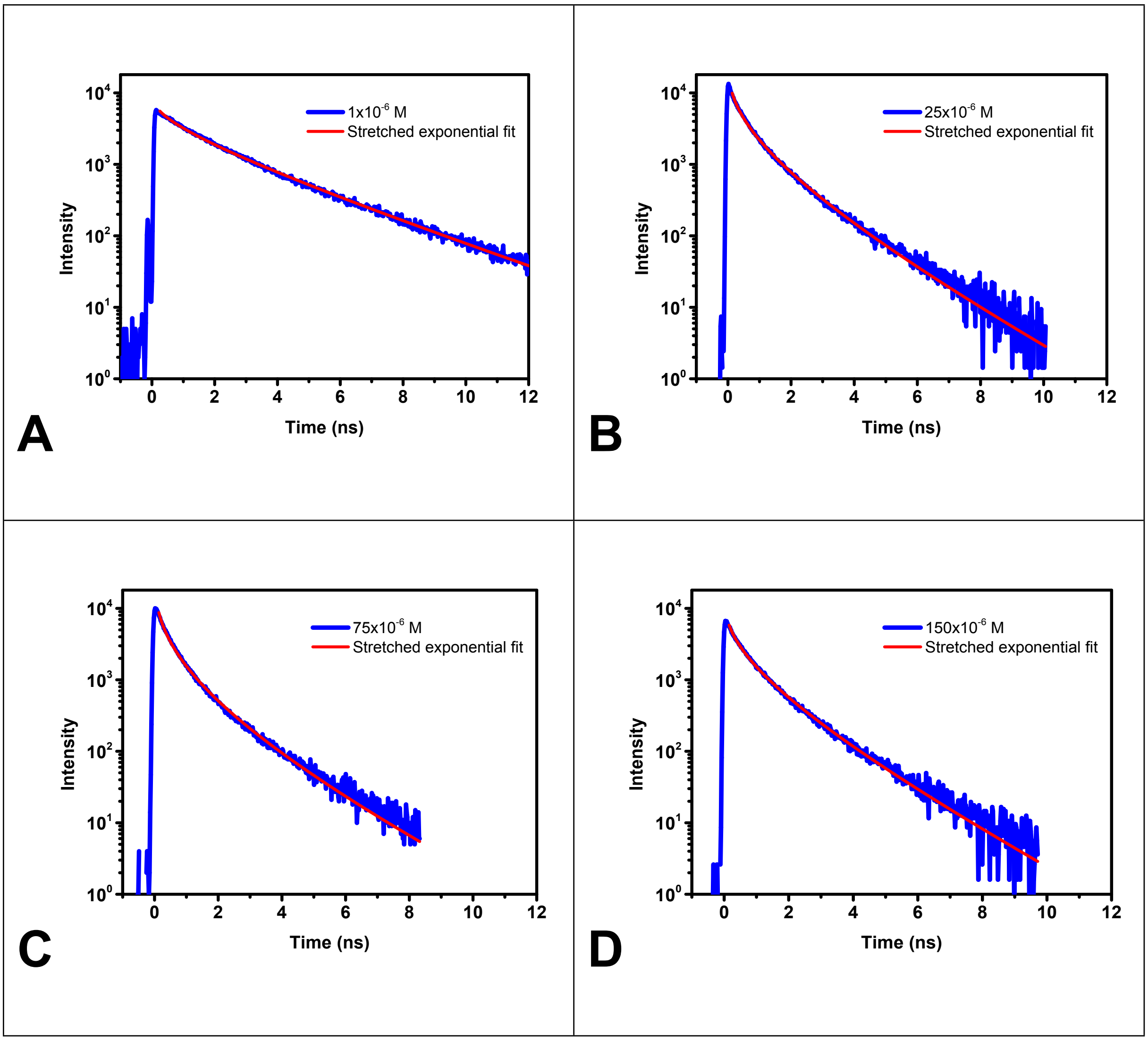

Figure S4 Fluorescence decays of TD2 adsorbed on hBN analyzed according to equation 5 with β fixed to 1/3. Excitation occurred at 500 nm and the decays of the emission were recorded at 580 nm. A) adsorption from a $1x10^{-6}$ M solution, b) adsorption from a $25x10^{-6}$ M solution, c) adsorption from a $75x10^{-6}$ M solution and d) adsorption from a $150x10^{-6}$ M solution.

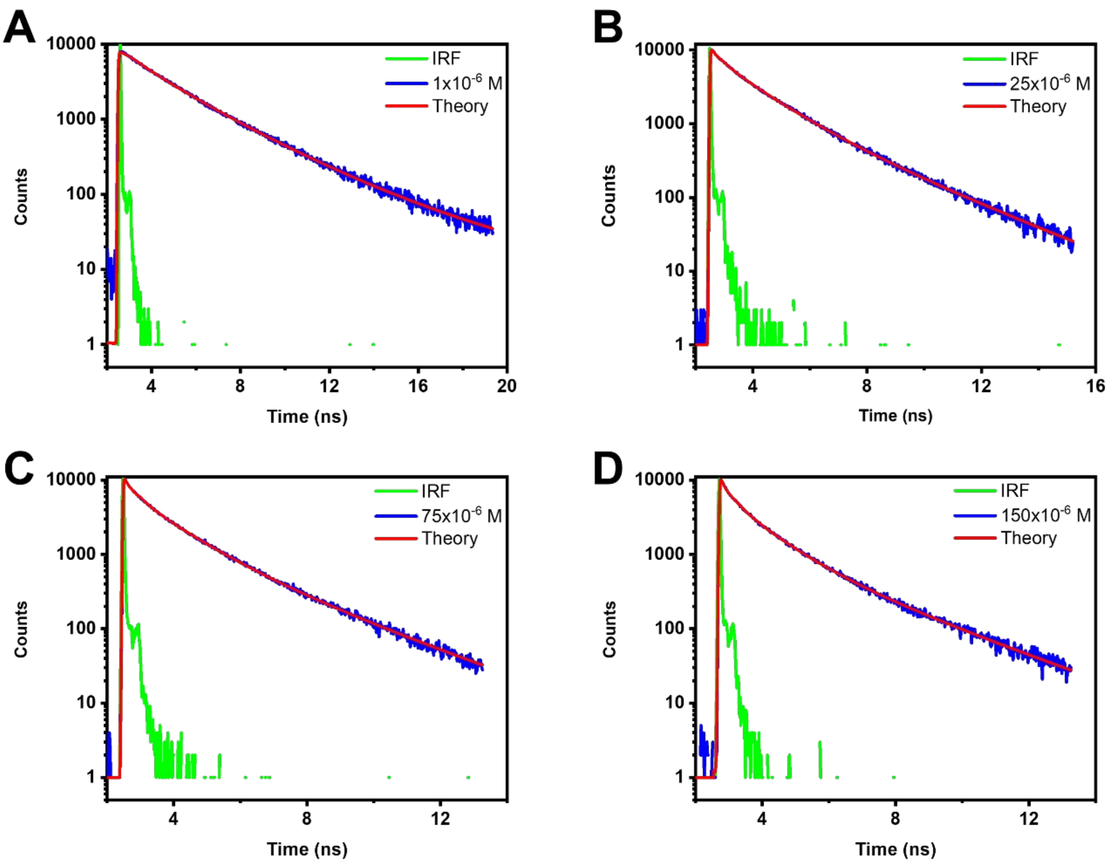

Figure S5 Fluorescence decays of TD0 adsorbed on hBN analyzed as a sum of exponentials. Excitation occurred at 500 nm and the decays of the emission were recorded at 580 nm. A) adsorption from a $1x10^{-6}$ M solution, B) adsorption from a $25x10^{-6}$ M solution, C) adsorption from a $75x10^{-6}$ M solution and D) adsorption from a $150x10^{-6}$ M solution.

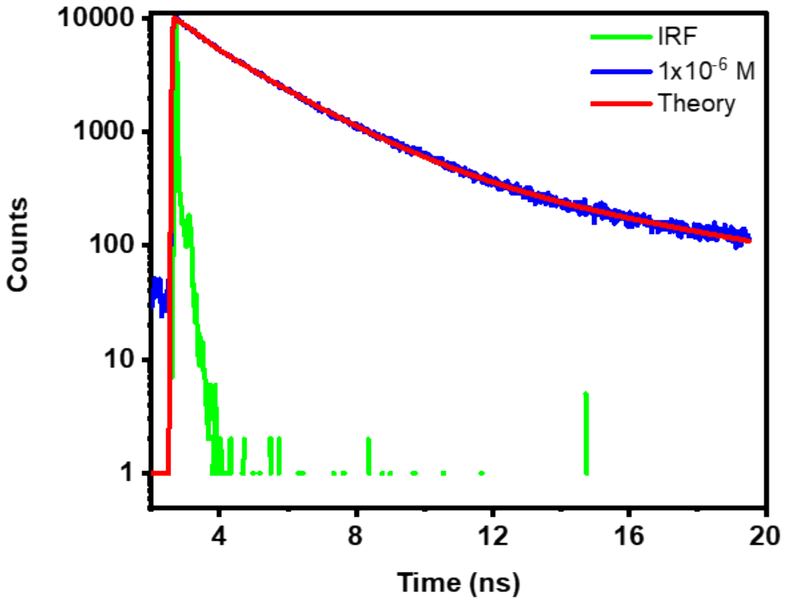

Figure S6 Fluorescence decay of TD0 adsorbed on hBN from $1x10^{-6}$ M solution analyzed as a sum of exponentials. Excitation occurred at 500 nm and the decay of the emission were recorded at 570 nm.

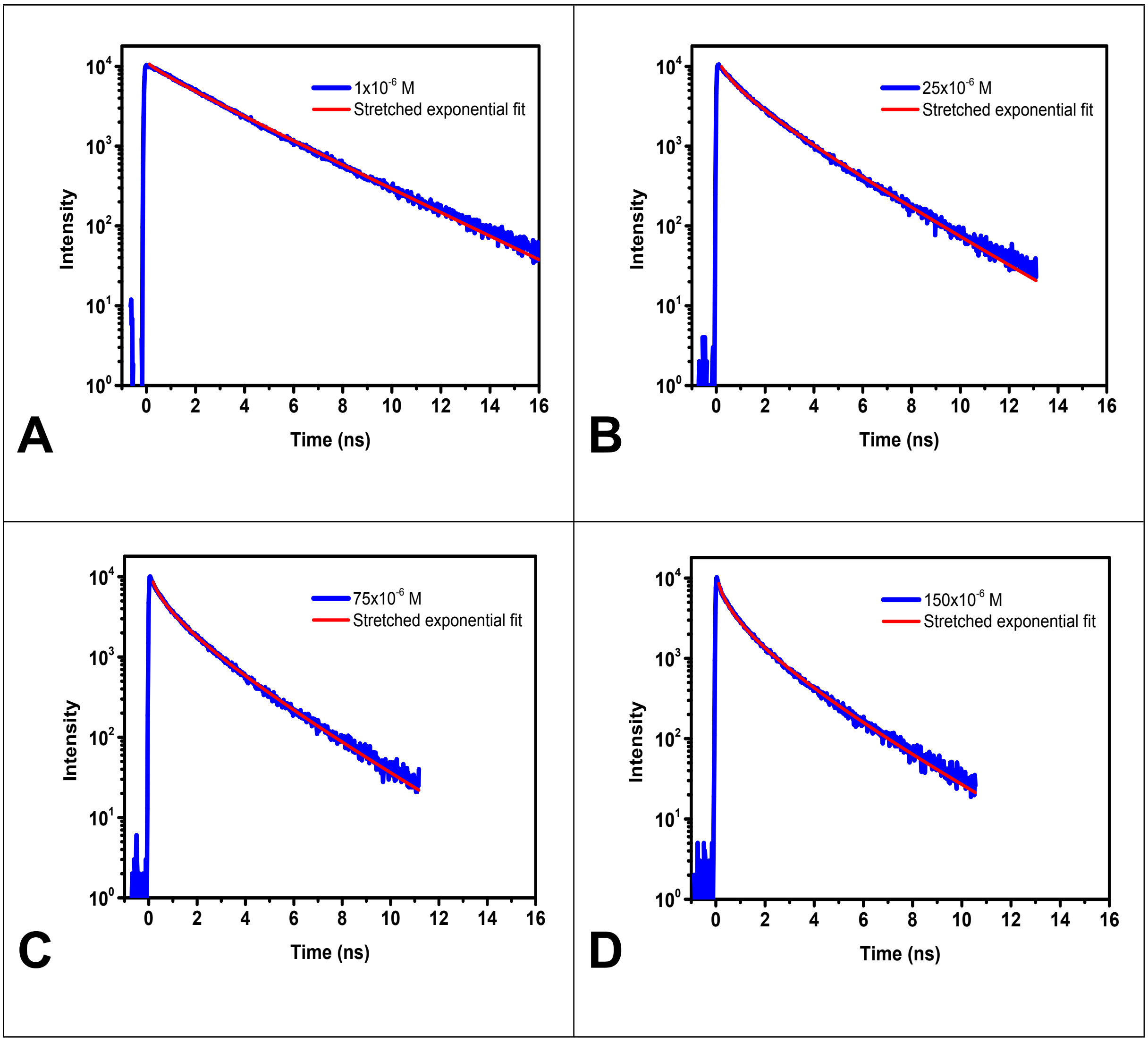

Figure S7 Fluorescence decays of TD0 adsorbed on hBN analyzed according to equation 5 with β fixed to 1/3. Excitation occurred at 500 nm and the decays of the emission were recorded at 600 nm. A) adsorption from a $1x10^{-6}$ M solution, b) adsorption from a $25x10^{-6}$ M solution, c) adsorption from a $75x10^{-6}$ M solution and d) adsorption from a $150x10^{-6}$ M solution.

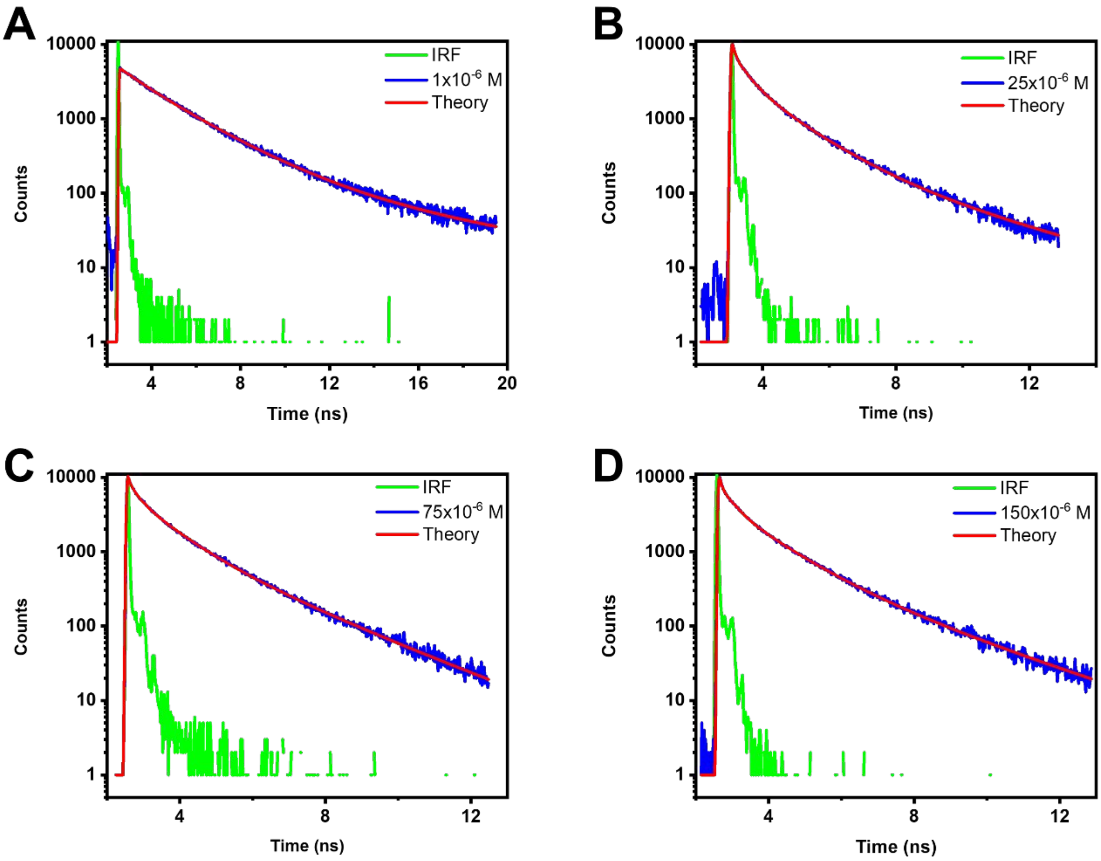

Figure S8 Fluorescence decays of THIATS adsorbed on hBN analyzed as a sum of exponentials. Excitation occurred at 500 nm and the decays of the emission were recorded at 580 nm. A) adsorption from a $1x10^{-6}$ M solution, B) adsorption from a $25x10^{-6}$ M solution, C) adsorption from a $75x10^{-6}$ M solution and D) adsorption from a $150x10^{-6}$ M solution.

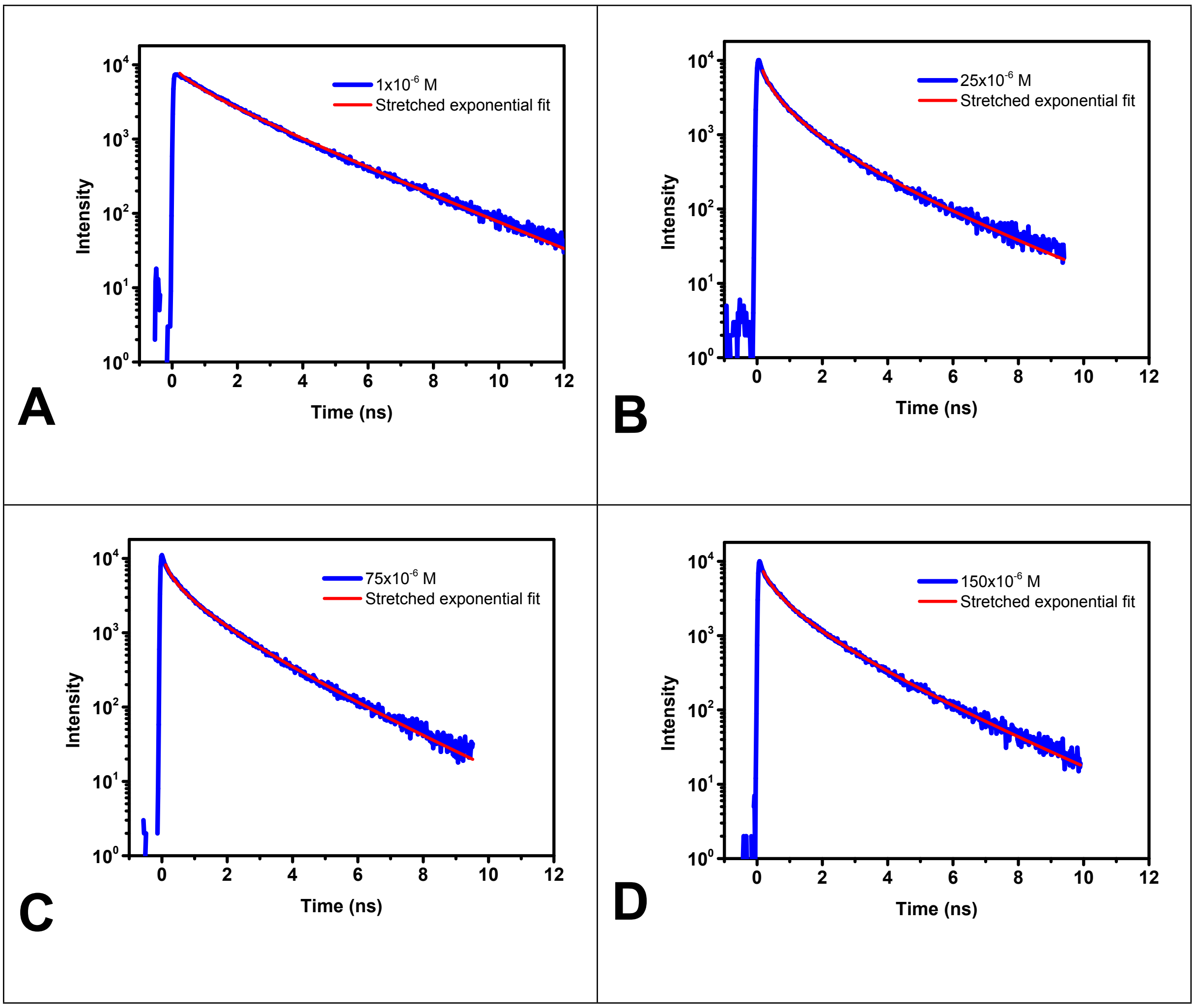

Figure S9 Fluorescence decays of THIATS adsorbed on hBN analyzed according to equation 5 with β fixed to 1/3. Excitation occurred at 500 nm and the decays of the emission were recorded at 600 nm. A) adsorption from a 1x10^{-6} M solution, b) adsorption from a 25x10^{-6} M solution, c) adsorption from a 75x10^{-6} M solution and d) adsorption from a 150x10^{-6} M solution.

Table S1 Contribution (p_1, p_2 and p_3) of the components p_1, p_2 and p_3, associated with the decay times (τ_1, τ_2 and τ_3 to the stationary fluorescence spectrum (in %) of TDC, TD2, TD0 and THIATS adsorbed to hBN.

C	λ_{Det}	TDC			TD2			TD0			THIATS		
(10^{-6} M)	(nm)	p_1	p_2	p_3	p_1	p_2	p_3	p_1	p_2	p_3	p_1	p_2	p_3
1	570	/	/	/	10.5	0.9	48.7	1.6	4.4	72.1	/	/	/
	580	0.6	3.9	61.8	30.4	2.8	44.8	3.3	26.4	64.5	1.4	11.5	72.5
	600	/	/	/	/	/	/	0.0	66.4	33.6	0.7	35.1	60.9
	620	0.0	0.0	70.0	0.0	0.0	37.0	0.4	66.6	33.0	1.7	38.3	55.5
25	570	/	/	/	10.5	26.4	50.4	1.4	9.5	54.6	7.6	21.6	46.3
	580	10.0	35.3	43.7	14.3	35.7	41.7	0.6	6.3	51.5	6.4	19.9	55.0
	600	/	/	/	/	/	/	0.0	5.8	50.2	8.6	23.5	52.2
	620	13.4	34.8	38.3	15.4	34.2	43.8	0.0	4.5	46.6	11.3	23.9	49.3
50	570	/	/	/	4.1	27.9	47.1	0.2	11.5	49.7	3.9	15.7	46.3
	580	8.8	30.7	49.2	5.1	27.7	47.6	0.2	9.3	48.7	3.8	22.1	52.6
	600	/	/	/	/	/	/	0.1	10.7	49.4	9.0	29.2	47.4
	620	7.1	41.2	47.2	4.4	27.8	47.1	0.1	10.8	45.6	7.1	27.1	49.6
75	570	/	/	/	8.1	31.4	37.9	2.2	14.6	52.6	2.2	15.8	42.9
	580	20.8	30.7	35.0	0.0	27.2	49.9	1.0	9.7	50.8	1.6	11.4	45.5
	600	/	/	/	/	/	/	1.1	10.9	49.7	1.5	12.5	45.9
	620	3.9	31.7	43.4	3.1	18.4	49.6	0.9	10.2	46.4	1.7	13.3	43.0
100	570	/	/	/	7.3	13.8	60.2	4.3	18.7	49.4	10.2	20.0	37.6
	580	7.0	30.0	42.2	12.8	34.1	39.9	4.1	18.7	52.7	10.2	24.0	38.7
	600	/	/	/	/	/	/	5.1	18.7	51.7	12.9	28.4	36.1
	620	5.2	30.5	44.1	11.6	34.1	42.9	6.2	19.4	49.5	20.3	32.2	33.2
150	570	/	/	/	4.0	19.0	62.6	3.4	16.3	49.1	10.1	20.3	42.3
	580	14.7	39.8	31.3	3.6	30.8	52.9	1.5	11.1	49.8	3.2	15.6	51.5
	600	/	/	/	/	/	/	2.4	10.9	48.6	2.8	16.1	53.5
	620	8.2	36.5	37.4	9.9	29.0	52.7	2.6	11.9	46.5	5.7	18.5	48.9

Table S2 Ratios α_2/α_3 and α_3/α_4 of the amplitudes of the intermediate and slow decaying components upon increasing the coverage level of TDC, TD2, TD0 and THIATS to hBN.

C	λ_{Det}	TDC		TD2		TD0		THIATS	
(x10^{-6} M)	(nm)	α_2/α_3	α_3/α_4	α_2/α_3	α_3/α_4	α_2/α_3	α_3/α_4	α_2/α_3	α_3/α_4
1	570	/	/	0.18	1.94	0.33	14.17	/	/
	580	0.21	3.23	0.62	3.24	0.70	33.07	0.31	18.71
	600	/	/	/	/	3.80	/	1.14	70.68
	620	1.5x10^{-5}	2.75	2.9x10^{-3}	0.93	3.88	/	1.37	46.62
25	570	/	/	1.23	10.26	0.80	3.27	1.49	5.35
	580	3.34	13.3	2.02	12.98	0.57	2.56	1.15	8.5
	600	/	/	/	/	0.53	2.35	1.43	9.21
	620	3.75	8.36	1.84	17.22	0.44	1.97	1.54	9.25
50	570	/	/	1.88	5.84	1.03	3.10	1.34	3.66
	580	1.88	17.25	1.85	6.30	0.79	2.80	1.65	6.62
	600	/	/	/	/	1.10	2.98	2.43	8.90
	620	2.31	25.38	1.87	5.87	1.00	2.53	2.15	8.26
75	570	/	/	2.63	4.50	1.07	3.77	1.73	2.65
	580	3.62	9.00	1.74	5.83	0.73	2.88	1.18	2.54
	600	/	/	/	/	0.85	2.85	1.28	2.75
	620	3.03	6.14	1.18	4.59	0.84	2.38	1.46	2.46
100	570	/	/	0.65	8.91	1.29	4.82	1.79	2.96
	580	2.38	5.19	2.43	8.40	1.21	5.79	2.09	3.62
	600	/	/	/	/	1.24	5.71	2.65	4.08
	620	2.32	5.62	2.26	10.45	1.34	5.36	3.27	5.89
150	570	/	/	0.87	10.76	1.25	3.98	1.97	3.62
	580	4.73	6.55	1.66	10.42	0.84	3.34	1.24	4.04
	600	/	/	/	/	0.84	3.21	1.23	4.52
	620	3.63	6.16	1.57	15.54	0.95	3.01	1.55	4.22

Table S3 The recovered values of fluorescence decay times (τ_i) in ns and corresponding normalized amplitudes (a_i) obtained from the fluorescence decays of TD2 adsorbed on hBN from solutions with different initial concentrations C (in mol/L). p_4 corresponds to the contribution of the component with the longest decay time to the stationary spectrum while <τ> in ns corresponds to the average decay time. The excitation wavelength was set to 500 nm.

C (x10^{-6} M)	λ_{Det} (nm)	τ_1 (ns)	α_1 (%)	τ_2 (ns)	α_2 (%)	τ_3 (ns)	α_3 (%)	τ_4 (ns)	α_4 (%)	p_4 (%)	χ^2	<τ> (ns)
	570	1.28	18.8		8.8		47.8		24.6	40	1.10	2.30
1	580		39.3	0.24	19.5	2.35	31.5	3.73	9.7	22	1.11	1.65
	620	0.003	0.01		0.14		48.2		51.6	63	1.36	3.06
	570		40.2		31.6		25.7		2.5	13	1.06	0.60
25	580	0.16	45.2	0.50	35.7	1.18	17.7	3.06	1.4	8	1.22	0.50
	620		47.7		33.2		18.1		1.0	7	1.20	0.49
	570		28.1		44.3		23.6		4.0	21	1.02	0.39
50	580	0.06	32.9	0.25	41.3	0.78	22.3	2.02	3.5	20	1.11	0.37
	620		29.7		43.3		23.1		3.9	21	1.06	0.38
	570		66.6		22.8		8.7		1.9	23	1.15	0.15
75	580	0.02	0.03	0.20	59.7	0.65	34.4	1.73	5.9	29	1.22	0.45
	620		48.7		25.2		21.4		4.7	29	1.04	0.28
	570		42.5		21.2		32.6		3.7	19	1.29	0.52
100	580	0.09	49.4	0.34	34.7	0.96	14.3	2.66	1.6	13	0.92	0.34
	620		46.3		36.1		16.0		1.6	11	1.06	0.36
	570		26.9		32.4		37.3		3.5	15	1.09	0.65
150	580	0.10	22.1	0.38	46.9	1.08	28.2	2.70	2.8	13	1.22	0.58
	620		44.7		33.0		21.0		1.3	8	1.17	0.43

Table S4 The recovered values of fluorescence decay parameters (B and τ in $ns^{-\beta}$ and ns) obtained from the fluorescence decays of TDC adsorbed on hBN from solutions with different initial concentrations C (in mol/L). The value of β was allowed to float but was kept linked over the decays obtained for different concentrations of the initial dye solution. The excitation wavelength was set to 500 nm. The fluorescence decays were obtained at 580 nm. Global χ^2 amounted to 1.46

C ($\times 10^{-6}$ M)	τ (ns)	B ($ns^{-\beta}$)	β
1	2.66	0.38	0.36
25	>100	3.71	0.36
50	3.29	2.71	0.36
75	20.83	3.63	0.36
100	8.20	3.63	0.36
150	20	4.24	0.36

Table S5 The recovered values of fluorescence decay parameters (B and τ in $ns^{-1/3}$ and ns) and extracted values of σR_0^2 obtained from the fluorescence decays of TD2 adsorbed on hBN from solutions with different initial concentrations C (in mol/L). The excitation wavelength was set to 500 nm. The fluorescence decays were obtained at 580 nm.

C ($\times 10^{-6}$ M)	τ (ns)	B ($ns^{-1/3}$)	σR_0^2	χ^2
1	3.46	0.89	0.99	1.79
25	2..29	2.33	2.27	2.29
1.8150	3.01	2.80	2.99	1.81
752.33	2.74	2.90	3.00	2.33
1001.82	3.38	2.97	3.30	1.82
1501.81	2.22	2.07	1.99	1.81

Table S6 The recovered values of fluorescence decay parameters (B and τ in $ns^{-\beta}$ and ns) obtained from the fluorescence decays of TD2 adsorbed on hBN from solutions with different initial concentrations C (in mol/L). The value of β was allowed to float but was kept linked over the decays obtained for different concentrations of the initial dye solution. The excitation wavelength was set to 500 nm. The fluorescence decays were obtained at 580 nm. Global χ^2 amounted to 1.55

C ($\times 10^{-6}$ M)	τ (ns)	B ($ns^{-\beta}$)	β
1	4.34	0.69	0.53
25	5.71	2.01	0.53
50	200	2.44	0.53
75	143	2.57	0.53
100	>100	2.48	0.53
150	3.61	1.76	0.53

Table S7 The recovered values of fluorescence decay parameters (B and τ in $ns^{-\beta}$ and ns) obtained from the fluorescence decays of TD0 adsorbed on hBN from solutions with different initial concentrations C (in mol/L). The value of β was allowed to float but was kept linked over the decays obtained for different concentrations of the initial dye solution. The excitation wavelength was set to 500 nm. The fluorescence decays were obtained at 600 nm. Global χ^2 amounted to 1.34

C ($x10^{-6}$ M)	τ (ns)	B ($ns^{-\beta}$)	β
1	3.38	0.18	0.61
25	4.62	0.76	0.61
50	11.23	1.28	0.61
75	7.58	1.11	0.61
100	>100	1.63	0.61
150	3.15	1.38	0.61

Table S8 The recovered values of fluorescence decay parameters (B and τ in $ns^{-\beta}$ and ns) obtained from the fluorescence decays of THIATS adsorbed on hBN from solutions with different initial concentrations C (in mol/L). The value of β was allowed to float but was kept linked over the decays obtained for different concentrations of the initial dye solution. The excitation wavelength was set to 500 nm. The fluorescence decays were obtained at 600 nm. Global χ^2 amounted to 1.41

C ($x10^{-6}$ M)	τ (ns)	B ($ns^{-\beta}$)	β
1	3.01	0.54	0.48
25	16.12	1.98	0.48
50	25	2.54	0.48
75	3.85	1.36	0.48
100	>100	2.73	0.48
150	5.03	1.57	0.48
